# Black titania an emerging photocatalyst: review highlighting the synthesis techniques and photocatalytic activity for hydrogen generation

**DOI:** 10.1039/d1na00477h

**Published:** 2021-08-31

**Authors:** Suman Sekhar Sahoo, Sriram Mansingh, Pradeepta Babu, Kulamani Parida

**Affiliations:** Centre for Nanoscience and Nanotechnology, Siksha O Anusandhan (Deemed to be University) Bhubaneswar-751030 Odisha India kulamaniparida@soa.ac.in paridakulamani@yahoo.com

## Abstract

The TiO_2_ semiconductor photocatalyst is in the limelight of sustainable energy research in recent years because of its beneficial properties. However, its wide band-gap and rapid exciton recombination rate makes it a lame horse, and reduces its photocatalytic efficiency. Recently, researchers have developed facile methods for lowering the band-gap, so that it captures a wide range of solar spectrum, but the efficiency is still way behind the target value. After the discovery of black titania (B-TiO_2_), the associated drawbacks of white TiO_2_ and its modified forms were addressed to a large extent because it not only absorbs photons in a broad spectral range (UV to IR region), but also modifies the structural and morphological features, along with the electronic properties of the material, significantly boosting the catalytic performance. Hence, B-TiO_2_ effectively converts solar energy into renewable chemical energy *i.e.* green fuel H_2_ that can ultimately satisfy the energy crisis and environmental pollution. However, the synthesis techniques involved are quite tedious and challenging. Hence, this review summarizes various preparation methods of B-TiO_2_ and the involved characterization techniques. It also discusses the different modification strategies adopted to improve the H_2_ evolution activity, and hopes that this review acts as a guiding tool for researchers working in this field.

## Abstract 2

Schematic representations of black TiO_2_ preparations and its modifications to achieve benchmark hydrogen evolution.

## Graphical abstract

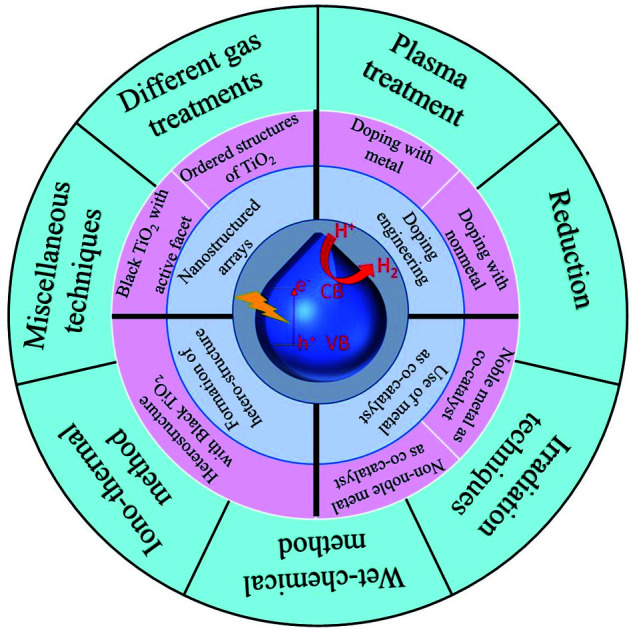

## 1. Introduction

The rapid and continuous growth of the human population and globalization are the major causes behind such extensive and meteoric use of non-renewable energy resources. As a result, the 21^st^-century world is facing its grievous consequences *i.e.* approaching energy crisis, fast depletion of fossil fuel reservoirs, and rising environmental pollution. This triggered and fostered the search for a renewable, economically feasible, and environmentally benign energy source that can substitute the long-standing fossil fuel economies in the upcoming years. In this context, molecular hydrogen as a zero-carbon footprint energy carrier is considered to be the most ideal and promising next-generation fuel because, (i) it has high energy density (140 MJ kg^−1^), (ii) produces water on combustion, and (iii) abundantly available feedstock. However, the industrial-scale production of hydrogen for various applications is satisfied through the stream reformation and coal gasification process, in which a substantial amount of anthropogenic greenhouse gases are released, causing severe ecological imbalance. Furthermore, the agreement signed by 196 countries on 2016 at the Paris conference made it a mandate for nations to adopt techniques for decreasing toxic gas emission to ultimately reduce the global warming index. Therefore, it is highly essential to turn to the use of renewable resources for the generation of clean and budget-friendly fuel. In this context, semiconductor-based solar powered hydrogen generation *via* water splitting is being intensively investigated because of its cheap and environmentally benign nature.

The typical artificial photocatalysis process involves three main steps: (i) absorption photon energy ≥ bandgap of semiconductor producing excitons, (ii) separation and diffusion of photogenerated electron–hole pairs from the bulk to the surface, and (iii) surface oxidation or reduction reaction caused by the photoexcited charge carriers, respectively.^1–3^ The efficiency of photocatalytic systems depends upon the thermodynamic and kinetic aspects of the above-discussed steps. For the semiconductor-based water-splitting reaction, the conduction band minimum must be more negative for hydrogen production (<0 V *vs.* NHE at pH = 0), and the valence band maximum needs to be more positive (>1.23 V *vs.* NHE at pH 0) for O_2_ generation. The historic experiment of Fujisima and Honda over a TiO_2_ photocathode towards H_2_ generation *via* water-splitting reaction has opened the gate for researchers to explore and find promising water-splitting photocatalysts that can substitute the conventional steam reforming/coal gasification process of H_2_ production.^4^ A large number of semiconducting materials have been developed and experimented in this research theme, which include noble metals, metal oxides, metal sulfides, metal phosphides, carbonaceous materials, layered materials, and metal nitrides. However, the catalytic efficiency (apparent conversion efficiency, quantum yield, solar to chemical conversion efficiency) of the main materials is way below the set value, owing to the low photon absorption ability, faster exciton recombination, slow charge diffusion, poor stability, and less exposed active reaction sites, which blocks the path of industrialization. Interestingly, the metal oxide-oriented systems show excellent catalytic activity and reusability compared to others. Specifically, TiO_2_ is being investigated rigorously because of its attractive and condition-favoring features, *i.e.*, unrivaled chemical stability, non-toxicity, feasible band edge potential, and low cost.^5–7^ In detail, TiO_2_ exists in three polymorphic forms, *i.e.*, anatase, rutile, and brookite, where Ti(iv) is coordinated with six oxygen atoms forming TiO_6_ clusters. Generally, in the anatase form (3.2 eV), the octahedron shares a corner at the (001) plane to form a tetragonal structure. Meanwhile, in the case of rutile (3.0 eV), the octahedron shares an edge at the (001) plane to have a tetragonal structure. However, for the brookite phase, the octahedron shares both edge and corner to form an orthorhombic structure.^8–12^ The anatase and rutile phases have been extensively studied for photocatalytic applications. In contrast, its natural phase *i.e.* brookite is less explored because of its preparation difficulties. The anatase form shows superior activity compared to rutile due to the efficient charge diffusion from the bulk to the surface and better exciton separation. However, the low light absorption capacity, faster charge carrier recombination, and slow electron–hole diffusion limits its widespread use. Therefore, to overcome the associated drawbacks and increase the catalytic efficiency of TiO_2_, several advanced techniques were implemented that include doping engineering, morphological tailoring, composite formation, and metal loading. This is no doubt that the he above-described methods increase the water reduction ability of TiO_2_. Still, the pursuit to commercialize has not been achieved, as few techniques are not budget-friendly, and some involve complex or tedious preparation steps. Furthermore, in some cases, the efficiency is still below the set mark. Hence, it is very demanding and challenging to design TiO_2_ with robust light-harvesting ability and low recombination to promote effective electron–hole pair separation capability. Furthermore, the optical and electronic properties of the solids largely depend upon the arrangement of bonded atoms, their distribution, and the lattice defects. Thus, by tuning them, the electronic and optical properties can be modified to ultimately improve the catalytic efficiency.^13–17^ Cronemeyer and co-workers have demonstrated the light absorption behavior of the white and hydrogenated TiO_2_ single crystals, and found that the hydrogenated one shows a broad window of photon absorption extending up to the IR region.^18^ Thereafter, a considerable amount of research had been performed over TiO_2_ to boost its optical properties.

In this context, Chen *et al.* demonstrated in 2011 a unique protocol for the synthesis of low bandgap TiO_2_ (*i.e.* 1.54 eV) by hydrogenation of TiO_2_ at 20 bar H_2_ pressure around 200 °C for 5 days, and coined the material as “black titania” (B-TiO_2_) due to its appearance.^19^ This discovery gathered global attention and encouraged the scientific community to begin studying this material for various photocatalytic applications. Generally, the black color of TiO_2_ is due to the presence of Ti^3+^, creation of oxygen vacancies, surface modification by hydroxylation, and formation of the Ti–H bond, leading to an effective alteration of the optoelectronic and catalytic properties. Furthermore, B-TiO_2_ shows enhanced photochemical activity as compared to pure white TiO_2_ because of its surface disorder and broad light absorption ability (UV to IR). Moreover, the synthesis of B-TiO_2_ involves two basic chemistry steps: (i) reduction of the +4 oxidation state to +3, and (ii) incomplete oxidation of the low valence state. The reported unique methodology leads to the creation of disorder in the surface of TiO_2_, which could enhance the photocatalytic performance, *e.g.*, H_2_ generation rate. The disorder engineering is due to the existence of an oxygen vacancy in the lattice framework of TiO_2_, which subsequently stabilizes the unusual oxidation state of titanium *i.e.* +3 state. Furthermore, the creation of the oxygen vacancy and subsequent stability of Ti(+3) in the lattice structure of TiO_2_ is the need of the hour for better light harvesting and improved photocatalytic activity. This can be done in various ways, such as metal and nonmetal doping into the lattice structure of TiO_2_.^5,6,20–26^

Along with the bandgap tuning, modification of the surface is another route to enhance the catalytic activity, as the reactions occur on the surface of the photocatalyst.^27,28^ In the concept of surface modification, Pan *et al.* prepared hydroxyl-rich TiO_2_ and observed a significant improvement in the hydrogen evolution rate, which is attributed to an increase in water dispersibility and decrease in the agglomeration of TiO_2_ crystals.^29^ Additionally, our group has made significant advancement and achieved remarkable catalytic efficiency in this photocatalytic water splitting area by developing and experimenting with a wide range of catalytic materials, including metal oxide, sulphide, phosphide, layered material (MoS_2_, LDH, g-C_3_N_4_), metal–organic framework (MOF), and quantum dots.^30–54^ The team has also reported an impressive H_2_ evolution rate over different TiO_2_-based photocatalytic systems, but we are yet to touch the set benchmark efficiency.^55–59^ This bottleneck motivated us to explore more on TiO_2_ (the standard material) that led us to B-TiO_2_, and started framing this review with the hope that this will help others working in the field. In this review, we have discussed the recent developments in the field of B-TiO_2_, including various synthesis methods for its preparation with details of the characterization of prepared materials justifying the proper formation of the material. We have also discussed different modification techniques adopted by researchers to further improve the catalytic efficiency of B-TiO_2_. Furthermore, the review elaborates on some recently reported black TiO_2_-based photocatalytic systems with commendable water reduction efficiency. Lastly, the summary section throws some light on the upcoming scientific master plans towards the development of promising B-TiO_2_-oriented photocatalysts, which will fascinate and encourage researchers and industry working in the water-splitting reaction and bring the dream of sustainable H_2_ production to reality.

## 2. Fabrication of black TiO_2_

**Scheme 1:**
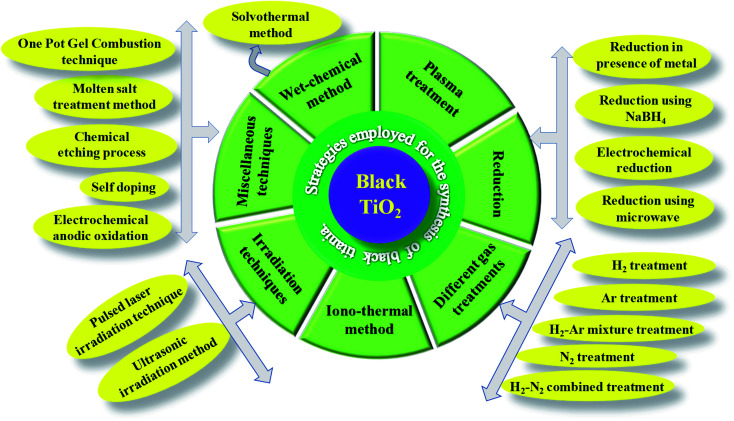
Different preparation techniques adopted for fabricating B-TiO_2_.

The facile fabrication of B-TiO_2_ has been a hard and challenging task, as it requires extreme pressure and temperature variation, as well as inert and vacuum atmospheric conditions. Hydrogenation has been considered as the most commonly followed technique in the near past for the preparation of B-TiO_2_. Moreover, it has been synthesized by various other methods, such as an inert atmosphere using argon only, hydrogen environment, nitrogen surrounding, hydrogen/argon treatment, nitrogen/argon treatment, oxidation, as well as reduction method, including reduction *via* metals like Al, Zn, Mg, reduction using NaBH_4_, hydrogenation using high and low-pressure variation methods, hydroxylation method, pulsed laser method, solvothermal method, ionothermal procedure, quantum dot method, and sol–gel method. Scheme 1 represents different methods adopted towards the preparation of B-TiO_2_, and the details of each synthesis procedure, and examples from the reported literature are discussed below.

### 2.1 Different gas treatment methods

#### 2.1.1 Hydrogen treatment

**Fig. 1:**
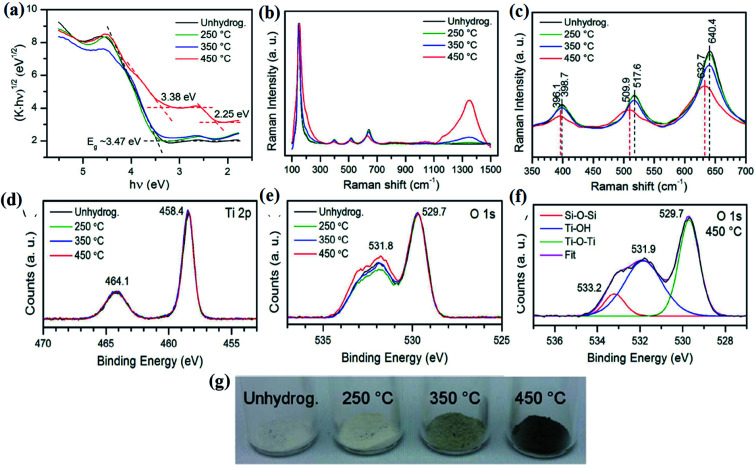
(a) UV-Vis DRS, (b and c) Raman, and (d–f) XPS spectra of H–TiO_2_. Reprinted with permission from ref. 60. Copyright 2012, American Chemical Society.

In 2011 for the first time, a B-TiO_2_ nanomaterial was synthesized by Chen *et al.* by hydrogenating a white TiO_2_ crystal at 20 bar pressure for 5 days, maintaining a temperature of 200 °C. The methods led to the formation of disorder-engineered nanophase TiO_2_, consisting of two phases *i.e.* a crystalline quantum dot as the core and a high disorder surface layer as the shell (both are of TiO_2_). The crystalline TiO_2_ quantum dot enhances the photocatalytic activity, whereas the surface of the disorder could absorb both visible and infrared radiation, enhancing the ability of the charge carrier trapping. The excess surface disorder could generate the mid-gap states having different energy distributions, as compared to the single defect crystals. The mid-gap states, also known as the band tail states, are those states that form a continuum within the conduction band edge, as well as valence band edge, causing a reduction in the bandgap. Moreover, the extended energy state is the dominant center for optical excitation and relaxation, as well as provides trapping sites for photogenerated charge carriers and inhibits the rapid recombination of excitons, which ultimately improves the overall photocatalytic efficiency. Furthermore, the Raman spectroscopy results of the disorder-engineered TiO_2_ showed new polarization peaks at 246.9, 294.2, 352.9, 690.1, 765.5, 849.1, and 938.3 cm^−1^, along with broader anatase peaks indicating the presence of a disorder active zone edge of the Raman forbidden mode of vibration. The XPS spectra are nearly identical for both white and disordered TiO_2_, suggesting no change in the chemical environment of Ti atoms, but with notable variation in O 2p spectra. The white TiO_2_ shows peaks at 530.0 and 530.9 eV, while the XPS spectra of the black TiO_2_ display a broad peak at 530.9 eV confirming the formation of the Ti–OH bonds. Furthermore, the optical analysis of the material by the reflectance and absorbance spectra suggested that the bandgap is narrowed (*i.e.* 1.54 eV) due to intra-band transitions. The authors also studied the change in the electronic and optical properties by DFT calculations, and found that the hydrogenation of anatase TiO_2_ shows sub-band formation, which reduces the bandgap. In brief, two sub-band states (at 1.8 and 3.0 eV) are observed for which the Fermi levels are present just below 2.0 eV. However, the midgap state at 3.0 eV is derived from Ti 3d, and the level at 1.8 eV is made of the O 2p orbital. More importantly, the presence of a hydrogen atom stabilizes the lattice disorder of B-TiO_2_.^25^ In another work, Leshuk *et al.* carried out hydrogenation of pristine TiO_2_ at different temperatures (250 °C, 350 °C, and 450 °C) under 20 bar pressure for 24 h, and observed the color change of TiO_2_ from white to black. They observed black coloration at 450 °C, and the noticeable change in optical absorbance of TiO_2_ calcined at different temperatures was analyzed using a Tauc plot. From the UV-Vis data, as shown in Fig. 1(a), the author found that the hydrogen treatment at a temperature below 450 °C shows a bandgap (*E*_g_) of 3.45 eV, which is greater than the band-gap of anatase TiO_2_, while the hydrogenation at 450 °C exhibits strong absorption with *E*_g_ = 2.18 eV throughout the entire visible range, which may be due to the inter-band states formed in TiO_2_ or due to the introduction of band tail states. It was observed that the crystallinity and structure of TiO_2_ are not affected by hydrogenation as confirmed from XRD, as well as morphological characterization. However, the broadening and shifting of peaks were also observed in the Raman spectra of B-TiO_2_ (Fig. 1(b) and (c)), having a flat hump at 1354 cm^−1^ for 450 °C hydrogenated TiO_2_, indicating the presence of Ti–H. Furthermore, the shifting and flattening of peaks are due to the presence of lattice disorders formed as a result of oxygen vacancies (V_O_). The Ti 2p and O 2p regions are found to be similar for both TiO_2_ and hydrogenated TiO_2_, as confirmed by XPS. In addition, the Ti : O ratio did not change significantly, while the O_OH_–O_L_ ratio increased very slightly for 450 °C hydrogenated TiO_2_. Apart from this, a slight increase in the concentration of OH at the surface was observed, which is probably due to the breaking of the Ti–O–Ti bonds after hydrogenation. The details are shown in Fig. 1(d)–(f). So, the author concluded that the strong visible light absorption is due to bulk V_O_ doping, rather than band edge shifting or surface modification.^60^

**Fig. 2:**
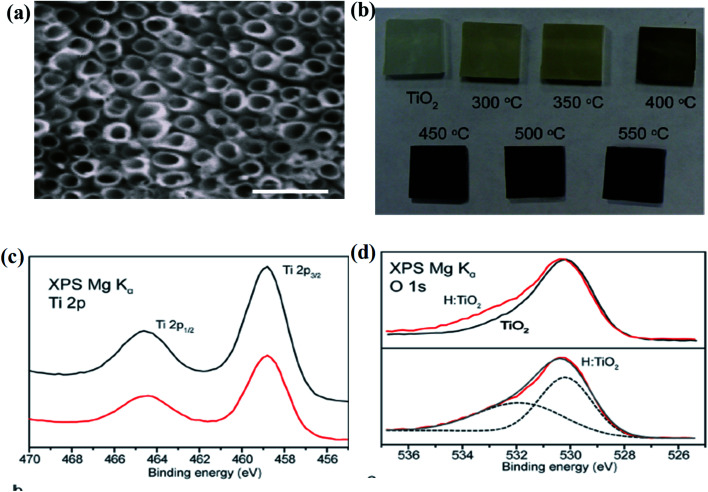
(a) SEM image of the electrochemically anodized TiO_2_ nanotube arrays, (b) the digital image of the neat and hydrogenated samples annealed at different temperatures, and (c and d) Ti 2p and O 1s XPS spectra of the parent TiO_2_ and H–TiO_2_ nanowires. Reprinted with permission from ref. 62. Copyright 2018, American Chemical Society.

Furthermore, Lu and the team fabricated B-TiO_2_ by hydrogenation of commercial P25 for 20 days at ambient temperature in a high-pressure atmosphere. The bandgap was almost reduced to 1 eV in comparison to P25 (3.2 eV), and the observed color change is due to the disorders formed at the surface. Hydrogenation at 35 bar hydrogen pressure generates a self-doped Ti^3+^ species with hydrogen atoms on the surface, as confirmed by XPS and EPR measurements. Both TiO_2_ and hydrogenated TiO_2_ showed OH stretching at 3400 cm^−1^ in the FTIR spectra, but the peak for OH stretching in hydrogenated TiO_2_ is quite broad. From the absorbance spectrum of B-TiO_2_ centered at 1.82 eV, the author suggested that the band-gap lowering is due to intra-band transitions.^61^ Wang *et al.* annealed nanowire arrays of rutile TiO_2_ in the open atmosphere for 3 h, followed by the treatment of hydrogen from 200 °C to 550 °C for 30 minutes, and noticed yellowish-green and black coloration of the material calcined at 350 °C and 450 °C, respectively. Hydrogenation of the TiO_2_ nanowire increases the donor density, which creates oxygen vacancies. From the XRD data, it was found that the intensity of the TiO_2_ peaks decreases with an increase in temperature during the annealing process, which is due to the increase in the defect density in the TiO_2_ crystal. Furthermore, Fig. 2(a) and (b) represents the SEM image of anodized TiO_2_ and digital images of pristine and hydrogenated materials, respectively. Additionally, XPS investigations of both materials (yellowish and black) are quite similar, showing that the hydrogenation process does not produce impurities in the lattice of TiO_2_. Besides, the hydrogenation process could create Ti–OH at the surface of B-TiO_2_ as confirmed from the XPS spectra, where two peaks at 530.2 and 532 eV are observed, as shown in Fig. 2(c) and (d). The hydrogen treatment has very little effect on the shifting of the valence band position, but the black color of TiO_2_ is due to the formation of defective sites in the lattice during hydrogenation. As the hydrogenation does not cause doping, the surface defect hence arises from oxygen vacancies and surface hydroxyl groups in TiO_2_. Moreover, the energy of the Ti–OH state is present just below the valence band of TiO_2_, causing extensive visible light absorption. In addition, hydrogenation creates oxygen vacancies and the energy of the oxygen vacancies lies at 0.75 and 1.18 eV below the CB of the hydrogen-treated TiO_2_ single crystal. Again, the absorption in the UV region is due to the transition of an electron from VB to CB, while the Vis and near IR absorption is due to the transition from VB of TiO_2_ to oxygen vacancy levels or from the oxygen vacancy levels to the CB.^62^

**Fig. 3:**
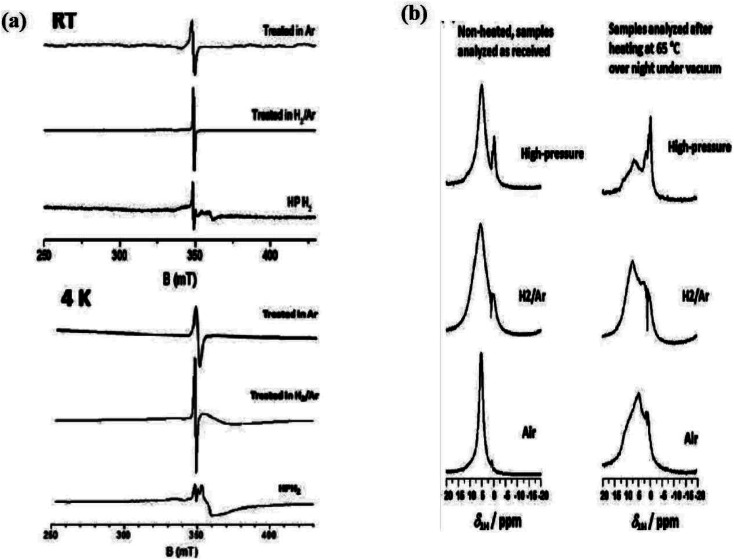
(a) EPR data and (b) NMR spectra of the TiO_2_ nanotube annealed in different atmospheric conditions (H_2_, Ar/H_2_ and high pressure H_2_). Reprinted with permission from ref. 66. Copyright 2016, Royal Society of Chemistry.

In another investigation, Sun *et al.* obtained hydrogenated B-TiO_2_ by incorporation of hydrogen into anatase TiO_2_. This hydrogen incorporation causes a color change from white to black, and makes the surface amorphous with a lattice *d*-spacing within the range of 10–30 nm. Interestingly, hydrogenation of the TiO_2_ (001) plane causes blue coloration, while hydrogenation of the TiO_2_ (101) crystal plane results in black coloration, indicating that the hydrogenation process greatly depends on the exposed facet. They observed that hydrogen incorporation does not change the bulk structure of the lattice. The Raman spectra of B-TiO_2_(101) showed anatase peaks without any broadening of the peak, while the shifting of the peak from 145 to 147 cm^−1^ is due to distortion in the lattice. Preferentially, hydrogen is added onto two coordinated oxygen atoms (O_2C_), along with a bond length of 0.97 Å with an oxygen atom, which is three coordinated (O_3C_). So, the hydrogen atom is tilted downward and forms two hydrogen bonds with O_3C_. Now, the hydrogen moves further downward, and the H–O_2C_ bond breaks to make H–O with an energy barrier of 1.49 eV at the sub-surface.^63^ Likewise, Naldoni *et al.* prepared B-TiO_2_ by heating amorphous TiO_2_ with hydrogen gas under an inert atmosphere, then underwent rapid cooling followed by heat treatment at 200 °C for 1 h, and reduction for another 1 h in a hydrogen atmosphere at 500 °C. The bandgap of white TiO_2_ is 3.15 eV, while that of the obtained B-TiO_2_ is 2.75 eV. The obtained B-TiO_2_ possesses 81% oxygen-rich anatase phase and 19% rutile phase, and both phases are crystalline with particle sizes of 23 nm for the anatase phase. If the temperature is increased above 700 °C, then the transition from anatase to the rutile phase occurs in the oxygen-rich regime. Meanwhile, under a reduction atmosphere, the transition temperature drops to 500 °C. Such annealing creates point defects in the crystal lattice of TiO_2_, creating oxygen vacancies (V_O_). The cathode luminescence spectra of B-TiO_2_ showed a broad band for three transitions *i.e.* two peaks at 2.63 and 2.36 eV for inter-gap states due to oxygen vacancies and one at 2.77 eV for the self-trap excitation emissions. The reduction of TiO_2_ generates oxygen vacancies and Ti^3+^ was present at the interstitial sites. In the above two cases, Ti is present at an excess amount compared to oxygen, and the reduction generates Ti^3+^. Furthermore, the absence of O_2_^−^ shows the non-existence of Ti^3+^ on the surface, while plenty are available at the bulk, which stabilizes the nanoparticle. The XPS data shows that the main absorption onset is present at 0.6 eV, while the maximum energy associated with the band tail shifted further towards the lower wavelength region, *i.e.*, at −0.3 eV.^64^ Additionally, Zhang and coworker synthesized mesoporous B-TiO_2_ nanotubes by the use of *Typha angustifolia*. The formed B-TiO_2_ shows sharp and intense characteristic peaks of the pure anatase phase having a high degree of crystallinity, with a *d*-spacing value of 0.35 nm and a pore diameter of 10 nm that corresponds to the (101) plane of anatase TiO_2_. Furthermore, the atomic force microscope (AFM) image showed several large, but thin surfaces-rough nanosheets with a thickness of nearly 4 nm for the sample annealed at 600 °C. The XPS spectra of mesoporous B-TiO_2_ are quite similar to those of mesoporous white TiO_2_, indicating the absence of Ti^3+^. However, the EPR spectra show a peak at a *g*-value of 1.97, indicating the presence of Ti^3+^ in the bulk. The combined effect of the disordered surface and band-gap narrowing increases the separation efficiency of the photogenerated electron and hole pairs, which ultimately increases the catalytic activity.^65^ Furthermore, Liu *et al.* prepared anatase B-TiO_2_ nanomaterials from the TiO_2_ nanotubes with a *d*-spacing value of 0.351 nm, which is very similar to hydrogen-treated TiO_2_ nanomaterials. The high-pressure hydrogen annealed TiO_2_ shows an EPR signal at *g* = [1.991, 1.974] at room temperature for the Ti^3+^ sites and a signal at *g* = 2.002 for the trapped electrons on the oxygen vacancies. Meanwhile, the ^1^H MAS NMR spectra show a peak at *δ* = 5.7 ppm for crystallographic water, which is present at the surface, and another peak at *δ* = 0 ppm was reported for the hydrogen atom present at the interstitial sites (Fig. 3(a) and (b)).^66^

**Fig. 4:**
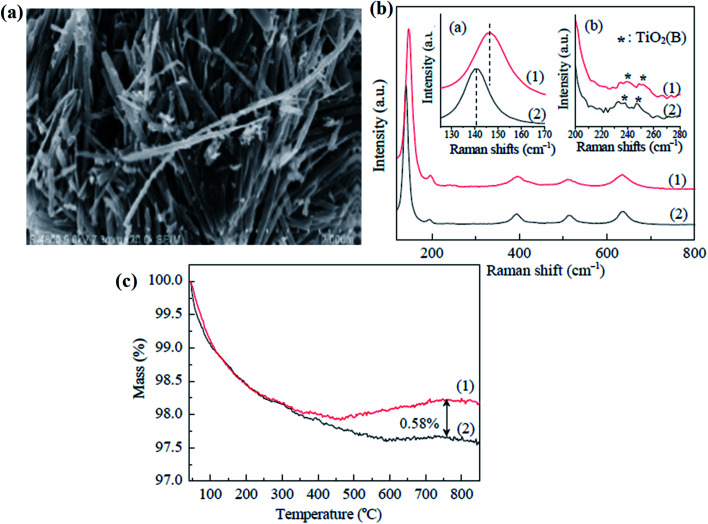
(a) FESEM image of the H–TiO_2_ nanofibers, (b) Raman spectra of the pristine and H–bicrystalline TiO_2_, and (c) TGA plot of the pure and H–TiO_2_ nanofibers. Reprinted with permission from ref. 67. Copyright 2015 Elsevier.

Similarly, Li *et al.* prepared nanofibers of black anatase TiO_2_. In this technique, potassium dititanate was treated with acid, followed by washing with water and then calcinated at 600 °C for 2 h under pure hydrogen, which causes oxygen vacancies at the bulk of B-TiO_2_. The oxygen vacancies are not localized and diffuse to the sub-surface from the surface. Moreover, the morphological structure of the nanomaterial is found to be fiber-like, having a uniform width of 0.2 μm and a length of nearly 1–5 μm (Fig. 4(a)). The surface area of the material is found to be 28.9 m^2^ g^−1^, indicating that hydrogenation has a negligible influence on the morphology. Again, the Raman vibrational mode gave six characteristic peaks, indicating the anatase phase, as depicted in Fig. 4(b). Additionally, the sample showed a blue shift in the Raman spectra, which illustrates that the reduced TiO_2_ contains defective sites. Furthermore, the XPS spectra of Ti 2p revealed two peaks at 459.4 and 464.9 eV, corresponding to Ti^4+^. So, it is concluded that the defects (oxygen vacancies) are not uniformly distributed on the TiO_2−*x*_ surface layers, and lie in the bulk rather than the surface. Furthermore, the TGA data (Fig. 4(c)) showed mass loss after 600 °C, and it is due to the loss of adsorbed water and hydroxyl groups. However, an increase in mass is observed for the oxidation of the sample. The author confirmed that the oxygen vacancy is 0.58%. The material showed a broad absorption range covering the entire visible region, and hence appears black.^67^

#### 2.1.2 Argon treatment

Doping of TiO_2_ with metal cations (like Ag, Sb, V, Fe) and non-metals (like S, C, I) introduces impurity states, which reduces the bandgap, enhancing its light-harvesting ability. Thus, the heating of metals at a very high temperature in an open atmosphere causes rapid oxidation. Therefore, a vacuum furnace is utilized for generating oxygen vacancy-induced TiO_2_, resulting in the formation of black TiO_2_. However, in this method for the cooling of the metal, a generally inert atmosphere is required. Specifically, the Ar atmosphere is capable of maintaining the furnace temperature below 200 °C.

**Fig. 5:**
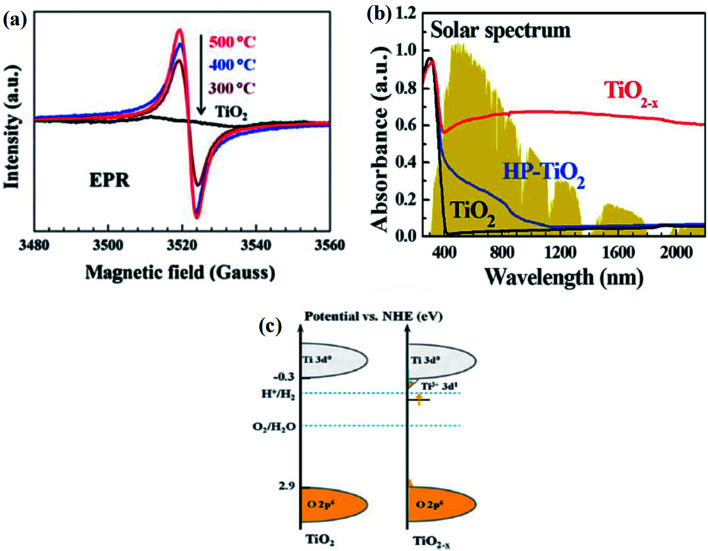
(a) EPR spectra of B-TiO_2_ treated at different temperatures and parent material, (b) optical absorption spectra of B-TiO_2_, (c) energy profile diagram of neat and reduced TiO_2_. Reprinted with permission from ref. 68. Copyright 2013, Royal Society of Chemistry.

Grabstanowicz *et al.* synthesized powder B-TiO_2_*via* a two-steps method, in which TiH_2_ was treated with H_2_O_2_, forming a slurry. It turned yellow in a vacuum and then dried at 100 °C, resulting in a powder. Furthermore, it was heated at 630 °C in an Ar environment for 3 h, and the black-colored rutile TiO_2_ was obtained. The XRD peaks confirmed that the intermediate formed is an amorphous powder, which upon calcination under an argon atmosphere at 530 °C, is converted into a rutile phase. With the increase in the calcination temperature to 630 °C, the formation of the rutile phase further increases. Additionally, it was observed by the authors that the self-doped Ti^3+^ rutile phase is more photoactive than anatase TiO_2_. The low-temperature EPR spectra (Fig. 5(a)) showed two *g*-values *i.e. g*_⊥_ = 1.975 and *g*_‖_ = 1.943, which were attributed to the axially symmetric lattice of Ti^3+^. In addition, the number of Ti^3+^ in the sample was found to be very large, *i.e.*, one out of 4.3 × 10^3^ Ti atoms were converted into Ti^3+^. Furthermore, the TGA study of the sample explained that the material is quite stable and the Ti^3+^ sites are located in the bulk, rather than on the surface, as shown in Fig. 5(b). The TEM images confirmed that the particles are highly crystalline. The average particle size for the argon-treated sample calcined at 530 °C is 200 nm, while that at 600 °C is 400 nm, which indicates that the particle size increases with an increase in the calcination temperature. The high concentration of Ti^3+^ generates a sub-band just below the conduction band minimum. So, the self-doped Ti^3+^ shows enhanced light absorption in the entire visible region extending from 400–800 nm (Fig. 5(c)).^68^

Adding more to the content, Myung and group prepared B-TiO_2_ by annealing the yellow TiO_2_ gel for 5 h at 400–600 °C in Ar flow. The material possessed both Ti^3+^ and Ti^4+^ with binding energies observed at 456 and 459 eV in the XPS spectra, and the ratio between the trivalent Ti^3+^ and Ti^4+^ was found to be 5.2 : 94.8 on the surface. The trivalent Ti atom was formed as a result of the highly acidic conditions, *i.e.*, pH = 2. Furthermore, the author confirmed that the formation of Ti^3+^ changes the structure, as well as the color of the substance. Again, the Raman polarization mode showed a broadening of peaks, suggesting the presence of oxygen vacancies. Interestingly, the XPS data does not show the presence of carbon, indicating that the color change is not due to carbon doping or carbon coating. Rather, it is because of the trivalent Ti and oxygen vacancy.^69^

#### 2.1.3 H_2_–Ar mixture treatment

**Fig. 6:**
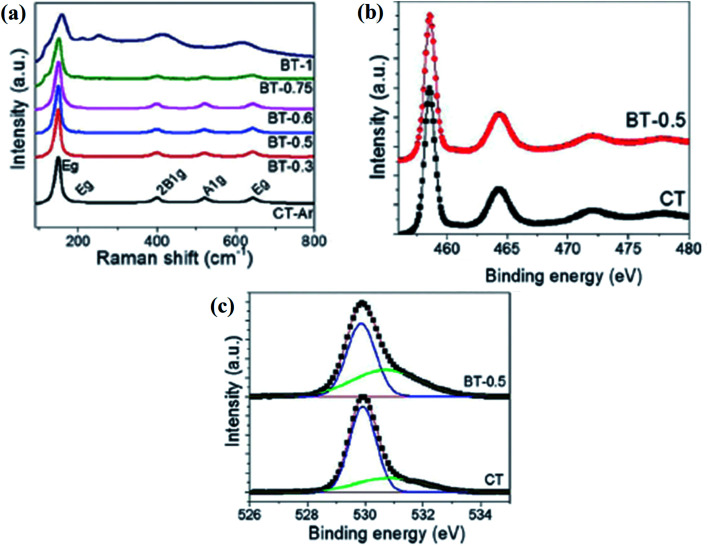
(a) Raman spectra, and (b and c) Ti 2p and O 1s high resolution XPS spectra of the prepared materials. Reprinted with permission from ref. 72. Copyright 2015, Royal Society of Chemistry.

The magnesiothermal process leads to modification on the surface, generating Ti^3+^ and oxygen vacancies. This results in a decrease in the bandgap, enhancing solar light absorption, and offering more active sites. Leshuk *et al.* separately reduced TiO_2_*via* hydrogenation using pure H_2_ and also under a mixture of H_2_ plus Ar, and a color change was observed in the resulting material. The hydrogenation is a highly temperature-dependent process and causes the breaking of surface bonds, which produces disorder in the crystal lattice, leading to the generation of band tail states, narrowing the optical bandgap, and allowing the catalyst to absorb photons in the visible region.^70^ Similarly, Lu and coworker applied hydrogenation to anatase TiO_2_ under an Ar–H_2_ mixed environment at 450 °C for an hour. Furthermore, the SEM and HRTEM images of TiO_2_ and hydrogenated TiO_2_ do not show any noticeable change in the morphology, which suggests that hydrogenation does not affect the structural framework. However, for the hydrogenated TiO_2_ nanotube arrays, there is the presence of a 2 nm thick amorphous layer, but the surface of the unhydrogenated TiO_2_ is devoid of this. So, hydrogenation created a disordered ∼2 nm thick layer covering the crystalline core. The XPS spectra of both TiO_2_ and reduced TiO_2_ are the same, but an extra carbon peak for both samples was obtained, which may be due to the exposure of the sample to the air during the experiment. The Ti 2p XPS spectra show peaks at 459.1 (Ti 2p_3/2_) and 464.9 (Ti 2p_1/2_) eV for anatase, but the absence of a signal at 457 eV indicates the absence of Ti^3+^ on the surface. This may be because Ti^3+^ is very unstable at the surface and converted back into Ti^4+^. However, at the bulk of the sample, three XPS peaks are observed at 462, 457 and 455 eV, indicating the presence of Ti^3+^, which was quite stable. The valence band of hydrogenated TiO_2_ shifted from 0.11 eV to 0.25 eV, narrowing the bandgap, which is due to the presence of oxygen vacancies in the crystal lattice. The preformed Raman analysis showed a broadening of Raman peaks for the oxygen vacancies produced by hydrogen treatment. The peak width depends inversely on the lifetime of the electron in a particular eigen state, *i.e.*, the smaller the lifetime value, the broader is the peak width. The FTIR spectra depict the characteristic peaks for the adsorbed water and OH group centered at 3446 and 1645 cm^−1^, while the Ti–O–Ti stretching vibration is visualized around 500–1000 cm^−1^, indicating that Ti is octahedrally coordinated in the lattice. Moreover, the oxygen vacancies generate additional electronic energy levels above the valence band, therefore lowering the optical bandgap.^71^ In another experiment, Sinhamahapatra *et al.* applied a magnesiothermal reduction strategy to synthesize B-TiO_2_ in the reduced form, where the concentration of Mg was varied under the hydrogen/argon atmosphere. Addition of Mg modifies the surface of TiO_2_, resulting in a color change of the material from white to grey, and then to black, with an increase in the concentration of Mg. The reduced TiO_2_ showed the characteristic Raman bands (Fig. 6(a)) of anatase TiO_2_ with the E_g_ band at about 148 cm^−1^, showing a blue shift along with peak broadening. The magnesiothermic reduction causes the loss of periodicity along with loss of symmetry of TiO_6_ on the surface generating oxygen vacancies. Additionally, the HRTEM images showed particle size around 10–20 nm with the *d*-spacing value of 0.35 nm for (101) anatase plane which is highly active for hydrogen generation. The Ti 2p XPS data shows band tailing towards lower binding energies indicating the formation of Ti^3+^ species. Further, the HR-XPS data of O 1s, a peak at 529.8 eV indicates the presence of the Ti–OH group at the surface which can also be correlated to oxygen vacancies or surface defects as shown in Fig. 6(b) and (c). It was found that the intensity of the peak increases with an increase in the concentration of Mg showing oxygen vacancies increase with the increase in the doping concentration of Mg.^72^

#### 2.1.4 Nitrogen gas treatment

**Fig. 7:**
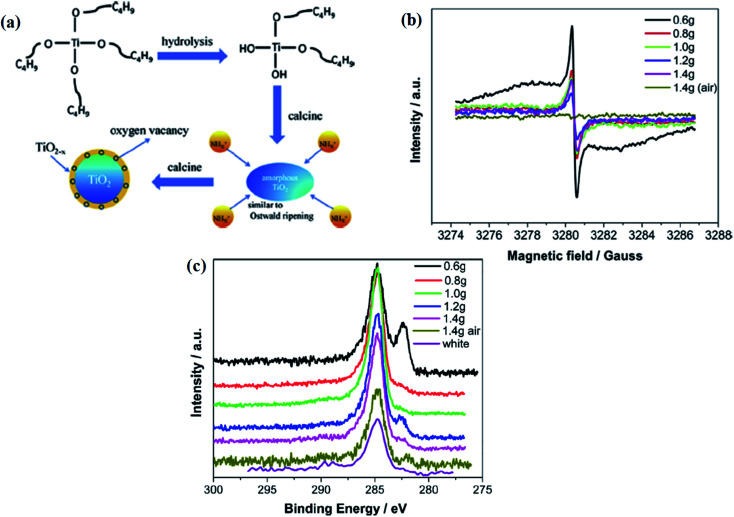
(a) Schematic representations of the formation mechanism of TiO_2_@TiO_2−*x*_, (b) EPR spectra, (c) C 1s XPS analysis of the white, black and yellow TiO_2_ after 60 s Ar^+^ etching. Reprinted with permission from ref. 73. Copyright 2016 Elsevier.

Doping with nitrogen is the most effective strategy because it causes bandgap narrowing. In TiO_2−*x*_N_*x*_, the shifting of the absorption maximum towards a higher wavelength is due to the mixing of the nitrogen 2p orbital with 3d of Ti^3+^, leading to the reduction of the bandgap energy. Under the N_2_ environment, Ti forms a bond with nitrogen because of the higher electronegativity of the N-atom, resulting in the higher binding energy of the Ti–N bond.^73^ In the work of Wei *et al.*, he proposed a one-pot synthesis for the fabrication of an anatase B-TiO_2_ core–shell nanostructure. In this method, colloidal TiO_2_ was taken as a precursor using tetrabutyl titanate with different weights of urea, ethanol, and distilled water in a water bath, followed by calcination at 500 °C for 3 h under a nitrogen atmosphere, and is schematically represented in Fig. 7(a). The experiment results in the same oxygen vacancy formation with the change in the oxidation state of Ti from +4 to +3. During nitrogen doping, the mixing of the N 2p and O 2p states occurs, which narrows the band gap, widening the photon absorption ability. Nitrogen doping generates Ti^3+^ and creates oxygen vacancies, which produce the surface disorders responsible for narrowing the band-gap (*i.e.* 1.38 eV). The author observed an EPR signal at *g* = 2.003, corresponding to the presence of Ti^3+^ and oxygen vacancies (Fig. 7(b)). Furthermore, the electronegativity of oxygen is higher than nitrogen. Therefore, the transfer of electrons occurs from nitrogen to oxygen, and then to the Ti-atom. As the electron density around the Ti atom increases, its orbital binding energy decreases. The binding energy of the sample containing 0.6, 0.8, and 1.2 g of urea was found at 400.78, 400.63, and 400.93 eV, respectively, for the Ti–O–N bonds represented in Fig. 7(c). Due to the shortening of the Ti–O bonds, the binding energy of oxygen increases further. As nitrogen is more electronegative than Ti, the electron density around the Ti atom decreases, increasing the binding energy of Ti. During the calcination process, the breaking of the Ti–N/Ti–O–N bonds occurs with the release of N_*y*_O_*z*_ or H_*m*_O_*n*_. Thus, the oxygen atoms attached to Ti were lost, creating oxygen vacancies.^73^

#### 2.1.5 Hydrogen–nitrogen combined treatment

Hydrogen is highly inflammable and under high temperature–pressure, hydrogenation is carried out, which is dangerous in the large-scale synthesis process. The hydrogen–nitrogen combined treatment is a less dangerous way of preparing B-TiO_2_. In this theme, Wu and his research team treated the TiO_2_ nanocrystal (derived from sol–gel technique) in a hydrogen–nitrogen mixture under different temperatures ranging from 110 °C to 210 °C. Furthermore, the obtained B-TiO_2_ has a crystalline core with a disordered surface layer possessing oxygen vacancies, which create localized states just below the conduction band minimum, resulting in a narrowing of the bandgap from 3.2 to 1.85 eV. Furthermore, the obtained XRD pattern showed the generation of the rutile phase from the anatase phase at 550–600 °C, as observed by the author. The rutile TiO_2_ is of bcc type in terms of its crystal arrangement with *a* = *b* = 4.58 Å and *c* = 2.96 Å. Meanwhile, the anatase form is also of bcc type, but with *a* = *b* = 3.78 Å and *c* = 9.52 Å. Moreover, the calcination atmosphere produces trivalent Ti atoms, oxygen vacancies, and surface defects in the crystal lattice. Interestingly, the concentration of oxygen vacancies within the prepared TiO_2_ annealed in the H_2_–N_2_ atmosphere is more than that calcined in an open atmosphere/oxygen-rich environment, indicating that the oxygen deficiency in the surrounding is the main cause of the vacancy, which further changes the material color, rather than the carbon doping.^74^

### 2.2 Plasma treatment method

The plasma treatment method is one of the most dangerous methods, as it causes severe burns upon contact with skin, and prolonged exposure can be lethal. Hence, this method of preparation is followed under utmost precautions and safety norms. The plasma processing technology is used to modify the surface physical and chemical properties of the materials. Upon variation of the electrical potential during plasma treatment; electrons, ions, and radicals are allowed to react and combine, causing surface modifications. Specifically, hydrogen plasma treatment is considered the best method because the photocatalyst (B-TiO_2_) prepared by this technique was reported to have the maximum solar light absorption capacity of about 83%. Wang *et al.* designed B-TiO_2_ using commercial P25 in a plasma furnace with an input power of about 200 W. The as-synthesized material shows broad absorbance in the visible region, and the obtained core–shell structure has a diameter of 20 nm with a crystalline core surrounded by an amorphous layer of 2 nm thickness stabilized by hydrogen. The enhancement in the absorption of solar light corresponds to the presence of an amorphous shell structure, which induces localized plasmon reactions on the surface. Again, the hydrogen plasma treatment reduces the localized Ti^3+^ sites, which act as the recombination center of photogenerated electrons and holes. Furthermore, the Raman spectra of the sample showed a shifting and broadening of peaks, confirming the disordered surfaces, and the signal at *g* = 1.957 in the EPR spectra appeared for the surface-adsorbed O_2_^−^ species. However, there is not enough evidence for the presence of a Ti^3+^ species in the core–shell of B-TiO_2_. The Ti 2p XPS data showed peaks at 458.5 and 464.3 eV for Ti(vi)=O and one extra band positioned at 457.1 eV for the Ti(iii)–H bond, while the O 1s XPS spectra showed a slightly broader peak at 531.8 eV consistent with the Ti–OH bond. The hydrogen plasma treatment has little effect on the position of the valence band. Additionally, the PL spectra of B-TiO_2_ depict a decrease in the peak intensity with an increase in the time of hydrogenation, specifying that the material has a very low recombination rate of photogenerated charge carriers, favoring enhanced photocatalytic activity. Extensive hydrogenation leads to the formation of additional vibrational modes at 3645, 3670, and 3685 cm^−1^ in the FTIR spectra representing Ti^4+^–OH. Moreover, the absence of a peak at 3710 cm^−1^, which corresponds to the terminal OH group, indicated the insertion of the H atom in the TiO_2_ lattice, breaking the Ti–OH bond. Additionally, the insertion of the H atom in the bridging sites results in a strong hydrogen bond with the OH group. So, a value at *δ* = 5.5 ppm is observed in the ^1^H NMR spectra, along with *δ* = 0.4 and 0.001 ppm for the terminal and internal hydroxyl groups, respectively. This again supports the above claim of the H atom in TiO_2_ lattice.^75^ In another breakthrough, Panomsuwan and his team prepared B-TiO_2_*via* a new plasma method, where plasma is produced within the water by placing two Ti electrodes and applying high-frequency pulses. The diffused reflectance spectra of H–TiO_2−*x*_ showed a strong and broad absorption in the visible and NIR range, having an absorption edge at 440 nm with a band-gap of 2.18 eV. Moreover, the presence of Ti^3+^ sites and oxygen vacancies is the main reason for the narrowing of the bandgap of the material. Furthermore, H–TiO_2−*x*_ has a perfectly spherical shape, and is distributed in a broad range in the bulk with a lattice fringe correlating to the rutile (110) plane. The prepared material exhibits a unique crystalline bulk with an amorphous surface layer. From the BET analysis, the author observed an increment in the surface area of H–TiO_2_*i.e.* 120 m^2^ g^−1^, which is because of the formation of rough surface layers. Furthermore, the XRD pattern confirmed that the nanomaterial is a mixture of rutile, anatase, and oxygen-deficient phases. However, the concentration of the rutile phase is quite high (only rutile *d*-spacing is observed), as the reaction occurs at high temperatures. The rutile and anatase peaks show broadening effects, which are due to the presence of oxygen vacancies, resulting in disorders in the lattice. Hence, it reduces the size of the crystallite. The Raman peaks for H–TiO_2_ are broader than P-25, and also showed a blue shift, confirming the oxygen vacancies and Ti^3+^ states. Due to some carbon contamination, the C 1s XPS peak was observed in the sample at 284.5 eV. The Ti 2p XPS spectra showed binding energies at 458.5 and 464.2 eV for Ti^4+^, Ti 2p_3/2_ binding energies at 457.2 and 456.2 eV and Ti 2p_1/2_ peak binding energies at 462.9 and 461.9 eV for the Ti^2+^ species.^76^

### 2.3 Reduction methods

#### 2.3.1 Reduction in the presence of a metal

**Fig. 8:**
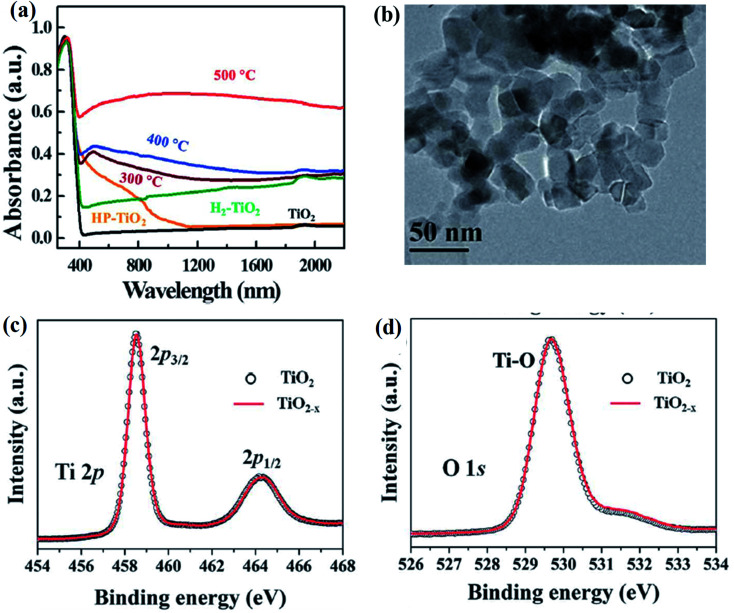
(a) Absorbance spectra of the TiO_2_ reduced at different temperatures, (b) TEM image of reduced TiO_2_, (c and d) XPS spectra (Ti 2p and O 1s) of the parent and reduced TiO_2_. Reprinted with permission from ref. 77. Copyright 2013, Royal Society of Chemistry.

Fabrication of B-TiO_2_ by metal reduction method is a cost-effective one, as it involves the use of low-cost and abundant metals (like Al, Mg, Zn) for the reduction process. In this regard, Wang *et al.* typically prepared B-TiO_2_ by pre-annulation of the TiO_2_ precursor with Al and Pt as the reducing agent at 800 °C for 6 h and 500 °C for 8 h, respectively, followed by post-annealing at 800 and 900 °C. The crystalline core and amorphous shell-structured TiO_2−*x*_ obtained after aluminum reduction underwent a color change starting from white to black. This was due to the vacancy of oxygen. The wide optical absorption was recorded ranging from visible to the IR region with an absorption edge at 440 nm having a bandgap of 2.18 eV, and the detail of this absorption is given in Fig. 8(a). The visible light absorption, along with the black color of the substance, is due to the presence of oxygen vacancies and the occurrence of Ti^3+^ species similar to that discussed in the above sections. The B-TiO_2_ photocatalyst has a preferably spherical shape with lattice fringes corresponding to the (110) plane of the rutile form, *i.e.*, the bulk region, which was confirmed from TEM analysis, and is represented in Fig. 8(b). So, the author suggested that B-TiO_2_ has a crystalline structure in the bulk and disordered surface layers possessing a surface area of 120 m^2^ g^−1^ (confirmed by BET analysis). The higher surface area is due to the rough adsorbing sites, and hence possesses higher catalytic active sites. The sample contains a mixture of rutile and anatase phase. However, due to high-temperature plasma treatment, the concentration of the rutile phase is much higher than the amount of the anatase phase. Furthermore, the material contains Ti^4+^, Ti^3+^ and Ti^2+^ states, as illustrated by XPS spectra (Fig. 8(c) and (d)), along with Ti^4+^–O, Ti^3+^–O and Ti^2+^–O species. Due to the band tailing, the band edge of the valence band maximum is shifted to 1.35 eV. This is likely for the presence of disorders in the surface, whereas the shifting of the conduction band maximum is for the oxygen vacancies and Ti^3+^.^77^

In another study, Lin *et al.* performed an Al reduction strategy to create oxygen vacancies on the amorphous surface over the crystalline core with Degussa P25 as a precursor (TiO_2_).^78^ Meanwhile, Zhi *et al.* prepared B-TiO_2_ by placing white titania and aluminum in a furnace at a pressure less than 0.5 Pa, and heating at 500–800 °C for 6 h under NH_3_ atmosphere. The treated sample was then cooled to get the B-TiO_2_ powders. The obtained nanomaterial has a diameter of 10–20 nm with crystalline anatase form. Furthermore, the EPR spectra of the sample with *g* values of 2.005, 2.004, and 2.003 ascertained the presence of Ti^3+^, nitrogen-doped TiO_2_, and single electron-trapped oxygen vacancies on the surface of B-TiO_2_, respectively. The presence of the Ti–N bond is again confirmed by the XPS spectra, where peaks are seen at 396.2 and 399.87 eV, respectively. Furthermore, the nitrogen adsorption–desorption curve showed a hysteresis loop in the *p*/*p*^0^ range of 0.9–1.0, suggesting that the material is highly porous with a surface area of 197 m^2^ g^−1^ (BET analysis).^79^ Likewise, Lin and co-workers synthesized B-TiO_2_ by the same aluminum reduction method, creating oxygen vacancies. Here, Al plays the role of a reductant, decreasing the partial pressure of oxygen to provide a driving force for the reduction of TiO_2_ at a lower temperature. The nano-crystal obtained has an average diameter of 25 nm with a unique core–shell structure. With the increased reduction temperature, the thickness of the disordered layer increases with a bandgap of 3.2 eV similar to the pristine TiO_2_. Additionally, an extra absorption shoulder peak is spotted at 500 nm. If the aluminum-reduced TiO_2_ is annealed at 800 or 900 °C, the color of the substance turned white due to the diffusion of Ti^3+^/oxygen vacancies, creating a dilution of color centers, which provides evidence for the presence of a heterogeneous core–shell structure. The blue shift and broadening of peaks in the Raman spectra were observed, confirming oxygen deficiency and the presence of an amorphous layer. Furthermore, the black TiO_2_ possesses a surface area of 42 m^2^ g^−1^, which is quite comparable with pristine TiO_2_ (43 m^2^ g^−1^).^80^

#### 2.3.2 Reduction using NaBH_4_

**Fig. 9:**
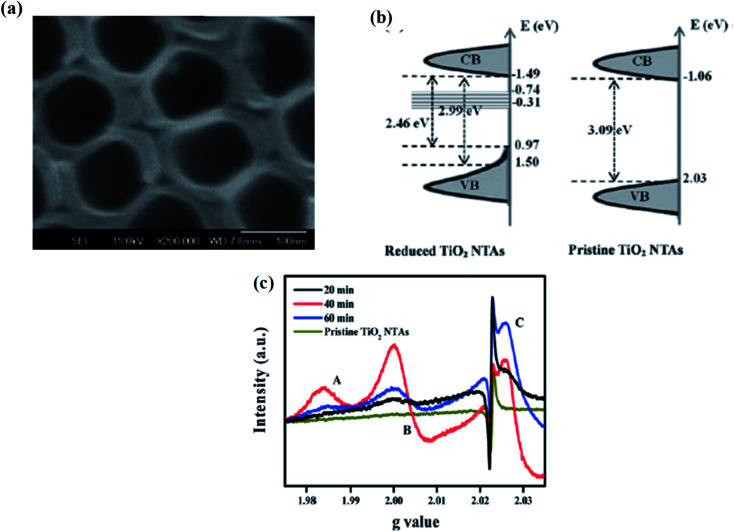
(a) SEM image of NaBH_4_-reduced TiO_2_ nanotube arrays. (b) Energy profile diagram of CV and VB. (c) EPR spectra of the TiO_2_ nanotube arrays reduced at different temperatures. Reprinted with permission from ref. 81. Copyright 2013, Royal Society of Chemistry.

TiO_2_ reduction using metal hydride (instead of hydrogenation) is comparatively safe and cost-effective, as NaBH_4_ type metal hydrides are less toxic and possess a strong reducing character. Kang *et al.* treated a solution of 0.1 M NaBH_4_ with prepared TiO_2_ nanotube maintained at room temperature for one hour to synthesize B-TiO_2_. The obtained B-TiO_2_ nanotube has an average length of 7 μm and an inner diameter of 100 nm with a highly porous structure (Fig. 9(a)). The improved photoresponse of the material is due to an increase in the charge separation as a result of the shifting of the Fermi levels towards the conduction band increasing the donor density, as well as electrical conductivity. The reduction process generates new surface defects, as well as oxygen vacancies and Ti^3+^. These oxygen vacancies are responsible for the enhanced visible-light photocatalytic activity. The reduction of the bandgap is for the presence of an electronic band located about 0.75–1.18 eV just below the conduction band, *i.e.*, the oxygen vacancy state. The details of the band arrangements are explained in Fig. 9(b). Furthermore, the density of state (DOS) calculation indicates that the valence band maximum suffers a blue shift to 1.05 eV with a bandgap tailing towards 0.1 eV, where the band edge position of the localized states are at −0.74 to −0.31 eV, causing enhancement in the photocatalytic hydrogen production rate. The EPR spectra gives the characteristic signals for the surface Ti^3+^, superoxide radical, and holes, along with a signal at *g* = 1.985 for the surface electron trapping sites in the case of the anatase form. The Ti^3+^ states adsorb oxygen from the atmosphere to form O_2_^−^. Hence, a signal at *g* = 2.03 (Fig. 9(c)) was obtained, indicating the presence of a large number of Ti^3+^ sites.^81^

**Fig. 10:**
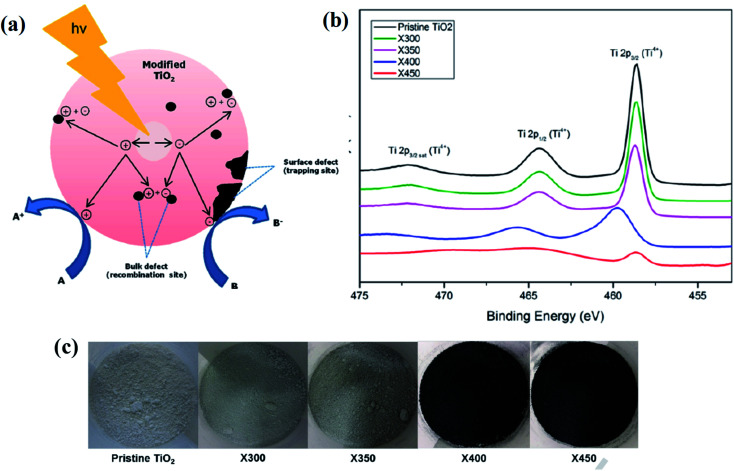
(a) Surface reaction mechanism, (b) Ti 2p XPS spectra of the parent and modified TiO_2_, (c) digital image of the reduced TiO_2_ samples synthesized by annealing at different temperatures. Reprinted with permission from ref. 83. Copyright 2016 Elsevier.

Furthermore, Tan and his team produced B-TiO_2_ by reacting P25 with NaBH_4_ in an Ar environment at varying temperatures *i.e.* 300 to 400 °C for an hour. During this reduction process, a sub-band is formed below the conduction band minimum due to the introduction of oxygen vacancies. With the increase in the concentration of oxygen vacancies, the shifting of the vacancy band occurs to a deeper region, which exhibits multiple bands in the range of 0.5–1.5 eV below the conduction band minimum. An interesting observation was made by the author, in this case, *i.e.*, as the reduction takes place in the solid-state so non-uniform reaction occurs, which results in the generation of uneven or inhomogeneous oxygen vacancies simultaneously. Hence, the valence band is placed deeper *i.e.* 1.2 eV below the conduction band minimum, while the valence band does not show any shifting. The XPS peaks showed the presence of Ti_4_O_17_, Ti_8_O_15_, and Ti_3_O_5_ with the rising of the valence band maximum up to about 1.0–1.5 eV, resulting in the reduction in the band gap energy. The oxygen vacancies create disordered TiO_2−*x*_ on the surface.^82^ Additionally, Ariyanti *et al.* synthesized defective B-TiO_2_ by reducing pure white TiO_2_ with NaBH_4_, where the mixture was calcined at 300–450 °C for 1 h. The systematic representation of the entire synthesis procedure is highlighted in Fig. 10(a). With the increase in temperature, the intensity of the XRD peaks decreases, indicating that at a higher temperature, the reducing agent attacks the long-range ordered surfaces of the TiO_2_ crystal, deforming the crystallinity of the lattice and producing oxygen vacancies. During the process of reduction, the generated active hydrogen from NaBH_4_ reacts with lattice oxygen, creating vacancies, and the excess electron may be located at the lattice of Ti to generate Ti^3+^. The XPS spectra (Fig. 10(b)) showed peaks at 529.8 and 531.3 eV, confirming the presence of Ti–O and OH bonds. In the XPS spectra of O 1s (OH), the peak area is higher for the modified sample, explaining that the disordered condition is more in the surface than the bulk. The excess electrons generated after hydrogenation lies in the oxygen vacancies, and hence act as a f-center and create mid-gap energy states, which are responsible for the absorption in the visible range. Along with the surface defects, the material also showed bulk defects, which may be composed of smaller size monovacancies. The annihilation rate of electrons and holes is reduced as the surface oxygen vacancies lowers the average electron density, thereby decreasing the rate of recombination. Fig. 10(c) shows the color variation of the prepared samples.^83^

Similarly, Fang *et al.* also reported on reduced B-TiO_2_ from TiO_2_ using NaBH_4_ through a single-step solvothermal treatment method. The author found that with an increase in the amount of reducing agent, the particle size decreases, along with the formation of oxygen vacancies and Ti^3+^. Importantly, the *d*-spacing value does not change after calcination, indicating that Ti^3+^ self-doping does not affect the unit cell dimension. The XRD pattern of the sample showed the self-doped Ti^3+^ and contains only anatase phase, while the precursor contains both rutile and anatase phase. This phase transformation may be caused by self-doping Ti^3+^ and oxygen vacancies. During this process, a large number of boron oxides are produced, which are adsorbed on the TiO_2_ surfaces as detected in the XPS spectra, but boron is not doped in the lattice because the characteristic peak at 187.5 eV for the Ti–B bond is absent. However, after washing with HCl, the peaks for boron disappeared. This is very essential because more color centers are exposed in this process, increasing the intensity of absorption. The reduction process also creates the Ti^3+^ color centers responsible for the higher visible-light photocatalytic activity. However, the Ti^3+^ generated on the surface is not stable, and can be easily oxidized by atmospheric oxygen as discussed in previous sections.^84^

#### 2.3.3 Electrochemical reduction method

**Fig. 11:**
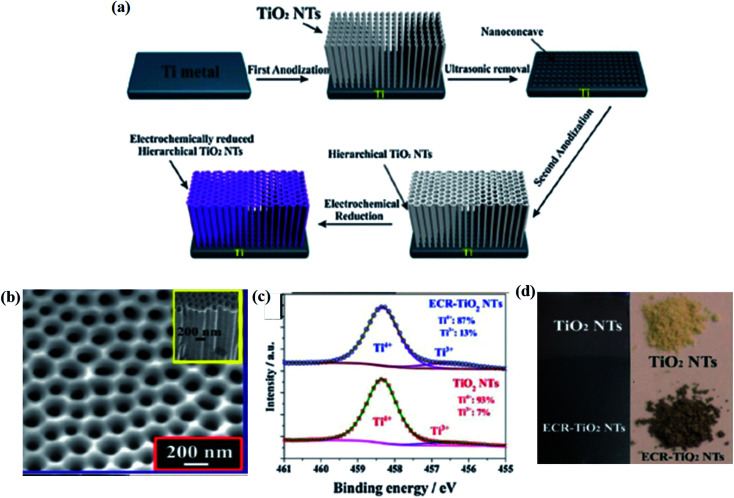
(a) Schematic representations of the anodization process, along with the electrochemical reduction procedure for the formation of self-doped TiO_2_ nanotubes. (b) SEM image of the electrochemically reduced nanotubes. (c) XPS spectra of Ti 2p_3/2_. (d) Digital image of the samples before and after the anodization process. Reprinted with permission from ref. 85. Copyright 2013, Royal Society of Chemistry.

In this electrochemical reduction process, one or more electrons are captured at the cathode with the passage of electricity through the electrolytic solution. Hence, electrochemical reduction plays an important role in the reduction of Ti^4+^ to Ti^3+^ for the synthesis of B-TiO_2_. In this context, Zhang *et al.* performed a two-step strategy for the anodization of the Ti metal to produce TiO_2_ nanotube arrays (TiO_2_ NTs), which act as the working electrode, Ag/AgCl as the reference electrode, and Pt mesh as the counter electrode for the electrochemical reduction of Ti^4+^ to Ti^3+^. In this three-electrode system, the TiO_2_ NTs were reduced under a negative potential biasing of about −0.4 V *vs.* RHE for 30 minutes using 1 M Na_2_SO_4_ as the electrolyte. It was allowed for the capturing of electrons *i.e.* reduction of Ti^4+^ to Ti^3+^, finally cleaned with deionized (DI) water, and dried in N_2_ atmosphere, as shown in Fig. 11(a). The self-doped Ti^3+^ generates a series of interstitial bands of Ti^3+^ with an energy range value from 0.27 to 0.87 eV below the conduction band minimum, enhancing the visible light absorption as the photoexcited electron travels from the VB to these sub-band states. The Ti^3+^ also increases the donor densities, enhancing the electrical conductivity. The reduced nanotube arrays (Fig. 11(b)) have a pore diameter range from 180–200 nm with hierarchical porous nanostructure with a thickness of 70 nm at the top layer, and the diameter lies in the range from 90–100 nm as observed from the morphological characterization. The length of the nanotube arrays was controlled to 2.4 μm. Furthermore, the XRD data showed a strong diffraction peak at 25.3°, explaining the highly crystalline anatase form of TiO_2_, along with small peaks at 2*θ* = 20.78°, 22.87°, 23.40°, 26.8° and 27.5° for the reduced TiO_2−*x*_ species like Ti_4_O_7_ and Ti_6_O_11_. The Ti 2p_3/2_ XPS spectra showed humps at a binding energy of 458.5 eV for Ti^4+^ and another peak at 456.6 eV for Ti^3+^ (Fig. 11(c)). Additionally, Fig. 11(d) depicts the digital image of the material before and after anodization.^85^

Furthermore, following this technique, Li and the group achieved B-TiO_2_ nanotubes by anodization of the Ti foil. Interestingly, the formed disorder-engineered TiO_2−*x*_ is highly stable for over one year, and possesses a narrow optical bandgap with excellent conductivity. The Ti 2p core XPS spectra are similar to the pre-annealed sample, which indicates that the Ti^3+^ may be present at the bulk, rather than the surface. The XPS spectra of O 1s ascertain the presence of acidic and basic hydroxyl groups, as peaks were observed at the binding energy values of 531.1 and 532.4 eV, respectively. Additionally, the peak located at 530.6 eV confirms the presence of lattice oxygen. The valence band maximum was positioned below the zero point by 3.1 eV for the doped sample. The upward shifting of the valence band maximum explains that the defect states are generated in the sample, which is due to the formation of donor states close to the valence band maximum. The color change is because of the insertion of the cation of smaller size inside the lattice under cathodic bias condition. Furthermore, the ultra-stability of the material is because of the presence of an organic solvent allowing H^+^ to diffuse into the deeper region of the TiO_2_ lattice, and also because H_2_O generates H^+^, which is difficult to reduce/oxidize; rather, it undergoes insertion to the lattice. The presence of oxygen vacancies increases the electrical conductivity, which facilitates the transfer of charge carriers, reducing the recombination of electrons and holes.^86^

#### 2.3.4 Reduction with manganese using microwave radiation

**Fig. 12:**
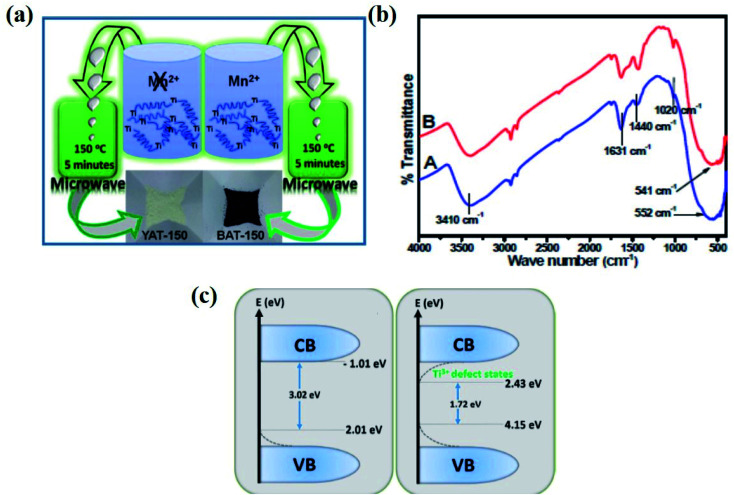
(a) Schematic representations of the following synthesis procedure. (b) FTIR spectra and (c) the density of states calculations of yellow and B-TiO_2_. Reprinted with permission from ref. 87. Copyright 2013, Royal Society of Chemistry.

The microwave-assisted synthesis is considered an eco-friendly, budget-effective, and facile method. Ullattil *et al.* developed B-TiO_2_ from the aqueous solution of titanium butoxide, manganese acetate by doping, and hydroxylation using microwave radiation. In short, the mixed sol was irradiated with microwave radiation at 150 °C at a stirring rate of 1200 rpm for 5 minutes, then dried at 80 °C, producing B-TiO_2_ and yellow TiO_2_. The detail of the diagrammatic representation of the synthesis procedure is shown in Fig. 12(a). The incorporation of Mn^2+^ with larger ionic radii compared to Ti^4+^ in the crystal lattice of anatase TiO_2_ created oxygen vacancies by forcing the lattice to withdraw oxygen and generate a new anatase peak. These generated oxygen vacancies lower the band gap, and hence shifts the absorption to a higher wavelength region of the electromagnetic spectrum. Meanwhile, in the absence of Mn, yellow TiO_2_ is formed. Moreover, the FTIR spectra (Fig. 12(b)) showed a broad band at 550 cm^−1^ for Mn^2+^ incorporation in TiO_2_, as well as the precursor. The bands for Ti–O–Ti, Mn–O, and Ti–OH vibrations are merged within the range of 400–900 cm^−1^. However, the peaks for OH stretching are present at 3420 and 1631 cm^−1^, confirming the presence of OH groups, and a peak at 1440 cm^−1^ was obtained for the Ti–O bond. Additionally, the presence of Mn^2+^ in an octahedral environment generates oxygen vacancies, creating lattice disorders and decreasing the bandgap to 1.72 eV. The Ti 2p_3/2_ and 2p_1/2_ XPS peaks are reduced to 457.65 and 462.65 eV due to oxygen vacancies, and the core O 1s XPS spectra are located at 528.65 eV for Ti–O–Ti, and also at 531.15 and 532.85 eV for the surface Ti–OH groups. The narrowing of the bandgap is due to the formation of sub-energy states, either above the valence band or below the conduction band (shown in Fig. 12(c)). Again, TEM analysis confirmed that the lattice fringe spacing of 0.339 nm corresponds to the (101) plane of anatase TiO_2_, and the size of the nanoparticle was 5 nm.^87^

### 2.4 Wet chemical methods

#### 2.4.1 Solvothermal method

This process involved the synthesis of a crystalline material using a high temperature and high vapor pressure environment using a solvent, and the precursors were kept inside an autoclave coated with a stainless steel material. The solvothermal method is generally a good synthetic method, where the shape and size of the material are handled in a controlled manner. However, the variation of the reaction conditions, such as the temperature, time interval, solvent, and precursor changed the property of the material. Shah *et al.* applied this facile method to synthesize B-TiO_2_ using l-ascorbic acid as a reducing agent. In this method, they treated different concentrations of ascorbic acid with TiCl_3_ in the presence of NaOH, which was heated at 180 °C for 12 h in an autoclave. The XRD analysis of defective TiO_2_ showed the anatase phase diffraction pattern with peaks at 25.4°, 37.9°, 48.1° and 53.1°. It was noticed that l-ascorbic acid does not affect the crystal structure of the sample. However, with an increase in the amount of ascorbic acid, the size of the particle decreases from 50 nm to 10 nm. Furthermore, the material contains a nano scroll-like structure with several layers of sheets of TiO_2−*x*_ with a spacing of 0.76 nm between two layers, and contains 56.4% of Ti and 28.8% of oxygen as verified by EDX analysis. The nanosheets are highly porous with an increase in the surface area from 64.56 m^2^ g^−1^ (white TiO_2−*x*_) to 188.75 m^2^ g^−1^ (brown TiO_2−*x*_) to 263.95 m^2^ g^−1^ (B-TiO_2−*x*_). All of the above TiO_2−*x*_ samples showed a peak at *g* = 2.003 in the EPR spectra, indicating the presence of trapped electrons in the oxygen vacancies on the surface of the material. However, it does not show any signal corresponding to the Ti^3+^ state. The enhancement of the photocatalytic activity is due to an increase in the concentration of oxygen vacancies, rather than Ti^3+^. All of the types of defective TiO_2−*x*_ depict a blue shift in the Raman spectra, confirming the breaking of the symmetry layers. The higher concentration of oxygen vacancies creates a new vacancy band just below the conduction band minimum of pure TiO_2_, which narrows the bandgap and enhances the photocatalytic activities, possessing a unique crystalline core with an amorphous surface layer.^88^

### 2.5 Ionothermal method

Using the ionothermal method, researchers prepared a single crystal by the use of an ionic liquid on the application of high temperature (400 °C) and pressure treatment inside an autoclave. Li and coworkers applied the ionothermal method for doping Ti^3+^ on TiO_2_ for better solar energy utilization. They prepared a buffer solution utilizing dihydrated lithium acetate, glacial acetic acid, and DMF, and then transferred the solution to the autoclave along with a cleaned Ti foil containing an ionic solvent (1-methyl-imidazolium-tetrafluoroborate) for 24 h that was maintained at 200 °C, followed by ethanol washing and drying for 12 h at 80 °C for producing the desired product. The sample is a single crystal TiO_2_ in anatase form with a particle size within 20–30 μm. Furthermore, the (001) and (101) exposed facets, along with (002) and (011) atomic planes having a lattice *d*-spacing value of 0.189 and 0.342 nm, again confirmed the anatase phase, respectively. If the calcination temperature is increased to 700 from 600 °C, it is completely transferred into the rutile phase. In the ^1^H NMR spectra, signals at *δ* = −0.18, 0.97 and 1.90 ppm indicate the occurrence of terminal hydroxyl groups. The EPR spectra showed a peak at *g* = 1.99–1.94 for the presence of the Ti^3+^ species. Besides, the XPS spectra of Ti 2p demonstrates that Ti^3+^ and Ti^4+^ coexist in the lattice. After 700 °C, Ti^3+^ is completely transferred into Ti^4+^ because of the oxidation effect. The Raman spectra highlights a principal peak at about 157.0 cm^−1^, which is the characteristic peak for the oxygen vacancy. With the increase in the calcination temperature, the XRD peaks suffered negative shifting, showing a decrease in oxygen vacancies. Oxygen vacancies are generated by the replacement of Ti^4+^ by Ti^3+^ ions. Upon adding more, the hydrogen atom, which is adsorbed on the Ti^3+^/TiO_2−*x*_ surfaces, generates mid-gap energy states that overlap with the edge of the conduction band to create band tail states. At a higher concentration of oxygen vacancies, the Fermi levels are placed closer to the conduction band tail, which allows the photogenerated electrons to exchange easily between the valence band and the conduction band under visible light irradiation.^89^

### 2.6 Irradiation techniques

#### 2.6.1 Ultrasonic irradiation method

**Fig. 13:**
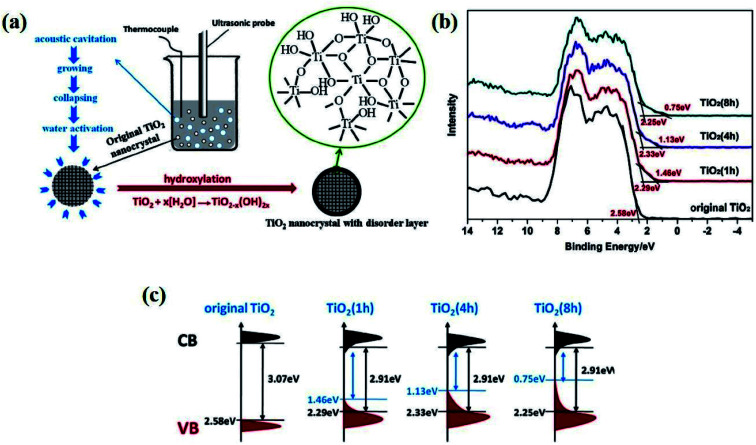
(a) Schematic representations of the surface disorders generated by the ultrasonication process, (b) VB XPS plot of the parent and TiO_2_ nanocrystals after ultrasonication, and (c) the energy profile diagram of the material for different time periods of ultrasonic treatment. Reprinted with permission from ref. 90. Copyright 2017 Elsevier.

Ultrasonic irradiation is a technique in which the liquid sample is agitated with ultrasonic waves at a frequency greater than 20 kHz. Here, the sound wave propagates into the sample, generating high pressure and low-pressure regions in the solution in a cyclic manner, resulting in compression and rarefaction of the medium, respectively, which is the main working principle of this method. Compression results in cavitation, whereas rarefaction creates small bubbles or voids in the liquid sample. *Via* this technique, Fan *et al.* synthesized B-TiO_2_ by prolonged ultrasonication of synthesized TiO_2_ sol for various time intervals, followed by drying at 80 °C (Fig. 13(a)). The TiO_2_ sol was prepared by mixing Ti(SO_4_)_2_ and NaOH in aqueous solution, and the prepared suspensions were then dried at 80 °C, while the other three suspensions were treated hydrothermally, followed by centrifugation and ultrasonic irradiation for 80 °C to obtain B-TiO_2_. The disorder-engineered hydroxylated TiO_2−*x*_ showed various degrees of blackness, depending upon the time of ultrasonic treatment. The XRD pattern for the material before and after ultrasonic treatment is quite identical, explaining the amorphous structure of the material. Furthermore, the Ti 2p_3/2_ XPS spectra (Fig. 13(b)) depict peaks centered at 464.2 eV for the presence of the Ti^4+^–O bond without any shifting or broadening of peaks, as the environment of the Ti atom before and after ultrasonic treatment is identical. Additionally, the O 1s XPS scan showed peaks at 530 (for Ti–O), 530.9, and 532 eV (for Ti–OH bonds). With an increase in the ultrasonic treatment time, the degree of hydroxylation of the material increases and is responsible for the black color of the material. The band positions of the materials prepared under different ultrasonication time periods is represented in Fig. 13(c).^90^

#### 2.6.2 Pulsed laser irradiation technique

This technique involved the utilization of a pulsed laser beam towards the targeted material placed inside a vacuum chamber, and irradiated with a laser beam obtained from various sources. By utilizing this pulsed laser beam method, Chen *et al.* synthesized visible active B-TiO_2_ nanospheres. In this technique, Nd:YAG pulsed laser beam was irradiated on both sides of the cuvette containing the TiO_2_ precursor, which was initially ultrasonically treated with distilled water. The color of the aqueous suspension was noticed as black after irradiation for 120 minutes, which was then filtered and dried at 80 °C for 12 h. The TiO_2−*x*_ nanospheres are polycrystals in nature (SAED) with a fringe separation of 0.218 nm corresponding to the (111) plane of the rutile phase (HRTEM). The powder XRD pattern of the precursor explained that it contains the anatase phase only. However, the percentage of rutile TiO_2_ increases as the duration of the laser modification increases. Furthermore, the O 1s XPS spectra showed peaks at 529.8 eV for lattice oxygen, 531.7 eV for the adsorbed oxygen, and peaks at 458.6 eV, along with peaks at 464.3 eV corresponding to Ti^4+^ 2p_3/2_ and Ti^4+^ 2p_1/2_, respectively. Another XPS band appeared at 457.5 eV for the Ti^3+^ 2p_3/2_, and that about 463.2 eV for the Ti^3+^ 2p_1/2_ species, specifically. Moreover, from the DOS calculation, it was verified that due to the laser irradiation, the valence band tail is bent upward about 0.4 eV and the conduction band tail downward for 0.7 eV, so the bandgap is reduced from 3.3 to 2.2 eV. Additionally, the surface Ti–H and Ti–OH bonds result in a blue shift of valence band maxima (VBM), creating sub-band energy states (formed due to Ti^3+^ or oxygen vacancies) just below the conduction band minimum without disturbing it. On the surface, the Ti^4+^ states get converted into Ti^3+^ by accepting electrons from oxygen atoms, and the oxygen atom leaves the surface, creating oxygen vacancies.^91^ In another system, Nakajima and coworker irradiated an ultraviolet pulsed laser beam for several minutes towards titania films to produce B-TiO_2−*x*_ in a vacuum. The material obtained by annellation at 700 °C, followed by pulsed UV treatment, showed a phase conversion to the rutile form anatase. The pulsed UV treatment creates oxygen vacancies in the lattice of TiO_2_, which is the reason behind the black color of the material, and it also generates Ti^3+^, which is the uncommon/unstable oxidation state of the metal. In the XPS spectra of Ti 2p_1/2_ and 2p_3/2_, the binding energies are observed at 464.3 and 458.5 eV for the presence of Ti^4+^. Furthermore, the O 1s XPS peak was observed at 529.8 eV for Ti–O bonds and 531.2 eV for Ti–OH bonds. This result suggests that the surface of the sample is oxygen-deficient and possessing oxygen vacancies, which increase the donor density and improve the charge transport property, along with shifting the Fermi level towards the conduction band. The oxygen deficiency and the crystallite size increase the carrier diffusion length, and hence decrease the rate of exciton recombination. The TiO_2−*x*_ film enhanced the light absorption in the visible region in comparison with white TiO_2_.^92^

### 2.7 Miscellaneous techniques

#### 2.7.1 One-pot gel combustion technique

The one-pot synthesis is a strategy for improving the efficiency of a chemical reaction, in which the reactant is converted to the desired product, following a single step in a reactor. Ullattil *et al.* carried out the one-pot combustion of the gel formed by the combination of titanium butoxide [Ti(OBu)_4_], diethylene glycol [DEG], and water, followed by heating for 2 h at 300 °C and then cooling, and again calcined at low temperature for the production of anatase B-TiO_2−*x*_ with a reduced bandgap of 1.51 eV. The obtained nanomaterials possess a higher concentration of surface defects, Ti^3+^, and oxygen vacancies with 33% increase in the photocatalytic activity compared to Degussa-P25. It possesses a high lattice strain (*c* = 0.334), which explains that the material contained a large number of defective sites. Furthermore, the XRD data show broad diffraction peaks, which explain that the B-TiO_2−*x*_ material has a small crystallite size, *i.e.*, 4.92 nm. The FTIR spectra also depict a broad peak of about 523 cm^−1^, indicating the presence of the Ti–O–Ti bond as a result of oxygen vacancies and peaks at 3430 and 1622 cm^−1^ for the stretching vibrations of the surface OH groups. The Ti 2p_3/2_ and 2p_1/2_ XPS scan shows humps at binding energies of 457.6 and 463.3 eV, confirming the generation of the Ti^3+^ species. The O 1s XPS spectrum shows a band at 528.7 eV for the Ti–O–Ti bond, and those at 531.2 and 532.9 eV ascertain the presence of Ti–OH and the free OH species, respectively. Due to band tailing, the valence band maximum is shifted to 0.62 eV. Furthermore, due to disorders extending below the conduction band, the conduction band minimum is shifted to 0.1 eV. Hence, the bandgap is decreased, extending the absorption towards the near IR region.^93^

#### 2.7.2 ZnCl_2_/KCl molten salt treatment method

In this method, Jijian *et al.* prepared B-TiO_2_ hexagonal nanosheets by the treatment of TiH_2_ with a molten eutectic mixture of ZnCl_2_/KCl, followed by grinding with ethanol. The so-formed powder was heated for 3 h at 400 °C, and then cooled and dried. The obtained amorphous B-TiO_2−*x*_ nanosheet matrix is made up of TiO_5_ pentahedral or TiO_6_ octahedra, which are linked with K–atoms. The nanosheets possess Ti^3+^ and oxygen vacancies at the surface, which shift the absorption maximum towards the IR region of the spectrum. Furthermore, the super stability of the structure is due to the presence of small crystalline hexagonal nanosheets embedded in the large amorphous nanosheet matrix. Additionally, the molten salt acts as the reaction medium, and speeds up the transfer of ions. The diffraction pattern confirms the rutile phase with a small amount of anatase phase. The hexagonal nanosheets are 350 nm thick, and are composed of tiny nanosheets of hexagonal shapes with a size of 10 nm, as seen from TEM analysis. The regular hexagonal nanosheets possess (110) and (101) lattice planes with a lattice fringe of 0.32 and 0.25 nm, respectively. Again, the angle between the two planes is 117, which indicates dislocation near the tiny rutile phase. Moreover, the EPR spectra possess a band at *g* = 2.02 for the presence of ˙O_2_^−^ on the surface, which is generated due to the oxidation of Ti^3+^ into Ti^4+^ in the presence of atmospheric oxygen. Furthermore, the Ti 2p XPS spectra for the 400 °C annealed TiO_2_ show bands at 457.4 and 463.4 eV, certifying the presence of Ti^3+^ on the surface of TiO_2_.^94^

#### 2.7.3 Chemical etching process

**Fig. 14:**
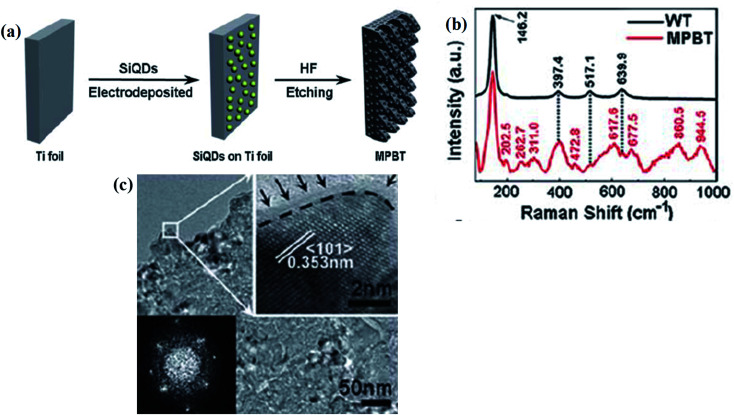
(a) Schematic representations of the procedure employed for the synthesis of mesoporous B-TiO_2_ nanocrystals, (b) FTIR spectra of white and black MPBT nanocrystals, and (c) TEM images of mesoporous B-TiO_2_ nanocrystals. Reprinted with permission from ref. 95. Copyright 2013, Royal Society of Chemistry.

This synthesis method includes the electro-deposition of Si-quantum dots on the surface of Ti-foil, and then chemical-etching using HF. Through this strategy, Huang and his research team prepared mesoporous B-TiO_2_ by chemical etching of deposited Si-quantum dots. First, they electrodeposited Si quantum dots on the surface of Ti-foil, and then subjected it to chemical etching by HF, as shown in Fig. 14(a). The oxygen vacancies and mesoporous structure (surface area of 762.67 m^2^ g^−1^ and pore size of 5 nm) resulted in the reduction of the bandgap and increment of the photocatalytic activity under visible light. The XRD pattern explained that the material contains purely anatase phase, and is highly crystallized without any impurity. Furthermore, the Raman spectra (Fig. 14(b)) present six polarization bands for anatase TiO_2_. However, new broad bands at 262.7, 311.0, 472.8, 617.6, 677.5, 860.5 and 944.5 cm^−1^ are observed. The broadening of peaks explained the presence of disorder or irregularity in the lattice, which is because of the activation of the zone edge or Raman forbidden modes of vibrations. The XPS spectra of O 1s showed peaks at 530.0 and 530.9 eV for the TiO_2_ nanoparticles and the presence of Ti–OH, respectively. Furthermore, the core XPS spectra of Ti 2p (disordered TiO_2_) is nearly identical with the precursor, explaining that the bonding environment of Ti has no change after the reduction process. The author found that the nanoparticles have an island-like structure containing only Ti and oxygen species. Again, the HRTEM images (Fig. 14(c)) confirmed that the product is highly crystallized with lattice fringes spacing of 0.353 nm, similar to the interplanar distance of the (101) anatase plane. Furthermore, the EPR spectra showed the presence of Ti^3+^ in a disordered rhombic ligand field of oxygen.^95^

#### 2.7.4 Self doping

This is an interesting strategy for the rapid synthesis of B-TiO_2_ with disordered surface and oxygen vacancies. Fan *et al.* synthesized B-TiO_2_ by adding titanium(iv) isopropoxide to a mixture of ethanol, 2-ethylimidazole, and hydrochloric acid with constant stirring, followed by heating at 500 °C. The reduced TiO_2−*x*_ possesses Ti^3+^ sites and oxygen vacancies, as confirmed by the below discussed characterizations. The presence of oxygen vacancies generates vacancy bands or mid bands just below the conduction band, which increase the visible light response for the nanomaterial. The obtained powder XRD pattern by the author explains that the material is a mixture of both rutile and anatase phases. Furthermore, in the EPR spectra, peaks are seen at *g*_*x*_ = *g*_*y*_ = 1.975 and *g*_*z*_ = 1.944, suggesting the presence of paramagnetic Ti^3+^ centers within the disordered rhombic oxygen ligand field. However, there is no Ti^3+^ on the surface, as it reacts with atmospheric oxygen to form O_2_^−^, as confirmed by the band at *g* = 2.02 (EPR). Moreover, the XPS spectra validate the presence of Ti^3+^ in the bulk of the lattice and the width of the band related to the concentration of Ti^3+^. Again, the DFT calculations confirmed that Ti^3+^ is present in the bulk and is responsible for the narrowing of the bandgap. The presence of oxygen vacancy sites also breaks the selection rule for the indirect transitions. Furthermore, enhancing the absorption of energy below the bandgap increases the photocatalytic activity in the visible and NIR region of the electromagnetic spectrum.^96^

#### 2.7.5 Electrochemical anodic oxidation method

**Fig. 15:**
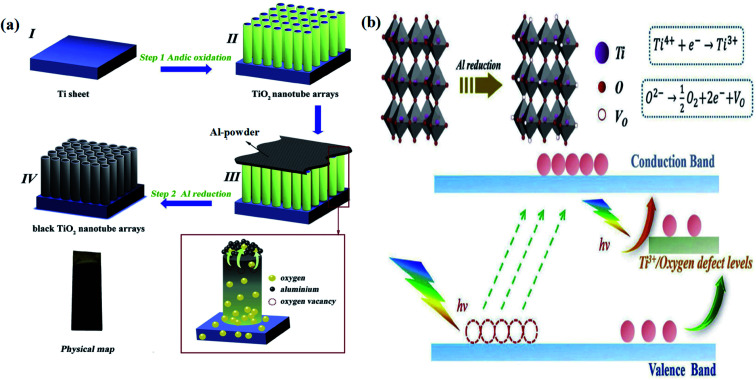
(a) Schematic representations of the synthesis procedure of B-TiO_2_ nanotube arrays, and (b) lattice structure of the material with its energy bands. Reprinted with permission from ref. 97. Copyright 2019 Elsevier.

Gao *et al.* prepared a B-TiO_2_ nanotube arrays film by electrochemical oxidation method (shown in Fig. 15(a)). In this method, TiO_2_ nanosheet arrays were cleaned with acetone, ethanol and deionized water, followed by anodization with NH_4_F and ethylene glycol at a potential of 50 V under room temperature for 1 h. It was then properly washed with deionized water, covered with Al-powder, and annealed (heating rate 3 °C min^−1^) at 250, 300, 450, and 600 °C for 2 h in Ar. In the aluminothermic reduction process, only oxygen vacancies are generated without any Ti^3+^, as oxygen is captured by aluminum from TiO_2_, producing B-TiO_2_ and extending the absorption of light towards the IR region. The enhancement in the absorption of light in the visible region is due to bandgap narrowing, while oxygen vacancies enhance the charge carrier density, transmission capability, and separation efficiency, which ultimately improves the catalytic efficiency. The FESEM images demonstrated that the nanotubes with regular ordered structures are vertically aligned and evenly distributed on the surface of the titanium foil. Furthermore, the nanotubes with open edges possess an inner diameter of 75–85 nm with 5–7 nm thicker walls, as visualized through TEM images. The space between the two adjacent lattice fringes is 0.35 and 0.238 nm for the (101) and (004) crystal planes, respectively. Furthermore, the XRD data illustrate that the nanotubes are highly crystalline with diffraction peaks for the (101), (004), (105), (204), (116), (220), and (301) planes of anatase TiO_2_. The aluminothermic reduced TiO_2−*x*_ showed a blue shifting and broadening of Raman peaks, which is due to the presence of oxygen vacancies, and the Ti 2p_3/2_ XPS data depict peaks at 458.5 and 464.2 eV for Ti^4+^–O bonds, indicating the presence of Ti^4+^, along with humps at about 457.6 and 463.2 eV, corresponding to Ti 2p_3/2_ for the presence of the Ti^3+^ sites. Additionally, for O 1s, three peaks are seen at the binding energy of 529.8, 531.6, and 533.1 eV, corresponding to the lattice oxygen, oxygen deficiency, and adsorbed oxygen species on the surface, respectively. The aluminum reduction process causes the doping of aluminum, confirmed by a sharp signal observed for Ti 2p. Furthermore, the peak at *g* = 1.99, as observed in the EPR spectra, implies oxygen vacancy sites on the material surface. The defects due to oxygen vacancies break the selection rule for the indirect transformation of TiO_2_, which enhances the photoactivity in the visible region by narrowing the bandgap. The lattice structure, along with the band position of the material, is given in Fig. 15(b).^97^

## 3. Color variation and structural identification

The extent of coloration depended upon the doping of impurities, hydrogenation, use of reducing reagents, application of metal and metal hydrides, pressure, and temperature variation. It has been noticed that the color changes from blue, green, yellow, grey, and black, depending on the reaction condition. For example, N doping to pristine TiO_2_ turned a yellowish coloration, whereas H doping was carried out for TiO_2_ for black coloration. The intensity of the black coloration depended upon the extension of sunlight absorption towards the IR region, the presence of oxygen vacancy, and Ti^3+^. Oxygen vacancies were identified by the measurement of D. C. Cronemeyer studies.

## 4. Strategies to enhance the photocatalytic hydrogen evolution activities of black titania

**Scheme 2:**
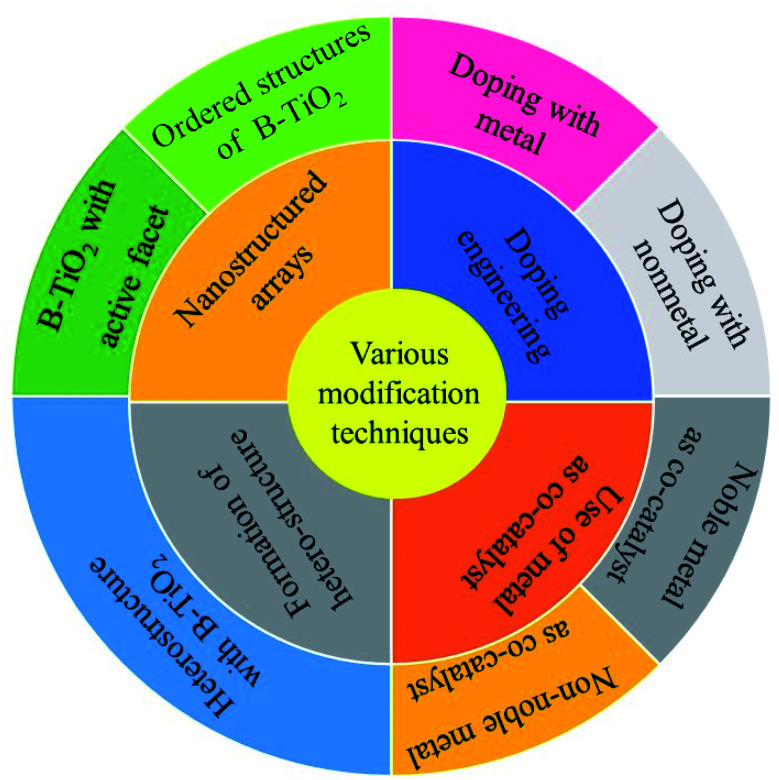
Schematic diagram shows various modification techniques for B-TiO_2_ to increase the hydrogen generation rate.

Since the first demonstration of B-TiO_2_ by Chen *et al.* in 2011, scientists have a vision that the optical absorption of TiO_2_ could be enhanced towards the more visible region by stabilizing Ti^3+^, and the subsequent creation of an oxygen vacancy in the lattice. However, the hydrogen generation by black titania is still challenging. Hence, various modifications, such as the doping of the metal and nonmetal into the TiO_2_ lattice, loading of the cocatalyst into its surface, and construction of a heterojunction with other effective semiconductors, have been studied by the researchers in the near past, and could be studied by the following heads. Scheme 2 shows different modifications of B-TiO_2_ to achieve an enhanced photocatalytic hydrogen evolution rate.

### 4.1 Metal doping

**Fig. 16:**
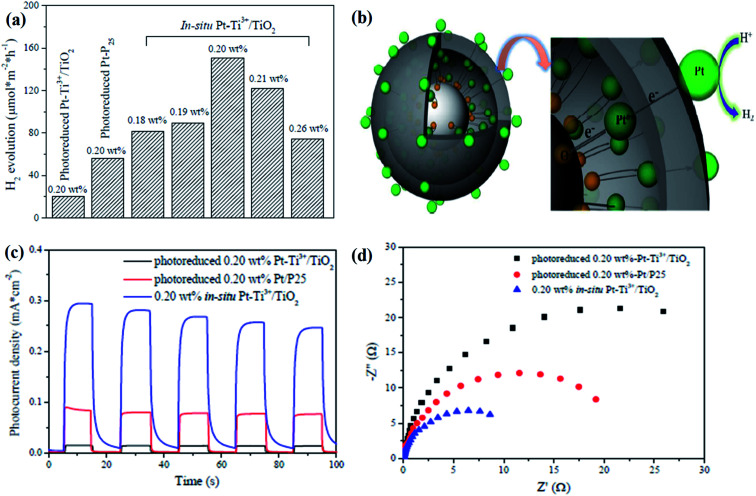
(a) Graphical representations of the hydrogen evolution rate by various Pt-doped TiO_2_ samples, (b) mechanism for the transfer of an electron from the bulk to the surface, (c) photocurrent response, and (d) Nyquist plot electrochemical impedance spectra. Reprinted with permission from ref. 98. Copyright 2016, American Chemical Society.

Doping a metal into the TiO_2_ lattice generally leads to the mixing of the atomic orbital of TiO_2_ (generally the 3d orbital of Ti) with the atomic orbital of the dopant. Lian *et al.* demonstrated Pt-doped titania *via* ionothermal process, where metallic Ti is dispersed in an ionic liquid containing LiOAc, HOAc and chloroplatinic acid aqueous solution under mild reaction condition. Pt entering the lattice of TiO_2_ could create oxygen vacancies by replacing Ti^4+^, which facilitates the electron transfer from the bulk to the surface of TiO_2_ leading to the reduction of protons into hydrogen. Furthermore, the oxygen vacancies and Ti^3+^ ion presence could allow for an environment for the insertion of Pt (0) and also Pt (*n*+) in the lattice, leading to a uniform distribution of the Pt valence state from the surface to the interior of TiO_2_. The *in situ* 0.20 wt% Pt-doped black TiO_2_ could produce a significant amount of hydrogen, which was 8 times higher compared to 0.20 wt% Pt–Ti^3+^/TiO_2_ obtained through the photoreduction process. The photocatalyst produces 151 μmol m^−2^ h^−1^ of H_2_ (as shown in Fig. 16(a)) with a quantum efficiency of 6.2%. The observed magnification in activity is attributed to the uniform insertion of Pt^*n*+^ ions, creating Pt–O linkage (from bulk to the surface) in the lattice framework of mesoporous TiO_2_. It is also due to the presence of ultrafine metallic Pt particles on its surface, which encourages the fast transfer of photoexcited electrons from bulk to the surface of the material *via* the Pt–O bond, which acts as a bridge for smooth flow. This observation is well explained in the photocurrent performance and impedance measurement. The possible electron transfer pathway from the bulk to the surface of the photocatalyst, along with the surface reaction, is shown in Fig. 16(b). In addition, Fig. 16(c) and (d) shows the transient photocurrent and impedance graph of the reported samples, respectively.^98^

### 4.2 Doping with the nonmetal

**Fig. 17:**
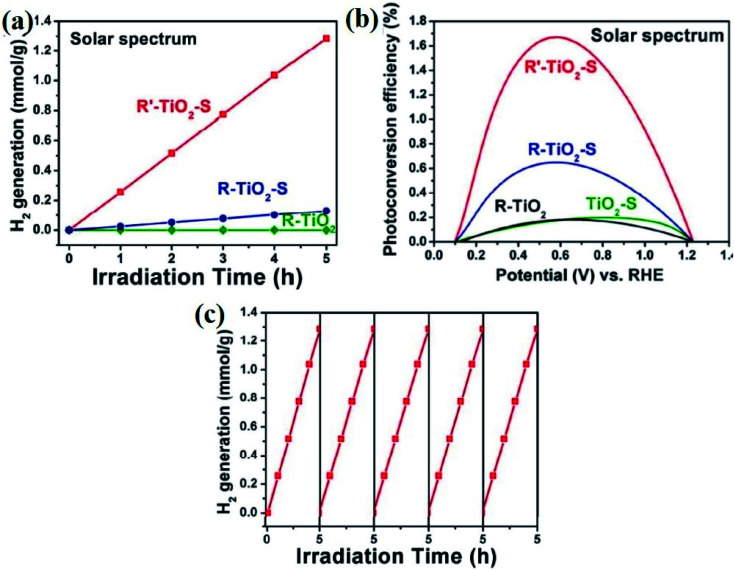
(a) Visible light-driven hydrogen generation rate of various doped TiO_2_, (b) photoconversion efficiency of various S-doped photocatalysts, and (c) stability data of the photocatalyst. Reprinted with permission from ref. 99. Copyright 2013, American Chemical Society.

Doping of metals is an efficient way for the modification of electronic band structures of semiconductor materials. However, doping with metals that show a poor photoresponse increases the impurity levels in the material, which promotes faster charge recombination, and also leads to a decrease in the thermal stability of the material. These shortcomings can be resolved by doping nonmetals into the lattice structure. In this context, a core–shell type nanostructured sulphur-doped TiO_2−*x*_ was prepared by Yang *et al.*, following a transformative cost-effective two step-method *i.e.* reduction through molten Al, followed by sulphidization by H_2_S, where rutile TiO_2_ is the core and sulfide is the shell (TiO_2−*x*_S), respectively. During the aluminum reduction process, a large number of oxygen vacancies are generated, which are occupied by S^2−^, resulting in a narrowing of the band gap by introducing S 3p orbitals and impurity caused by Ti^3+^. Furthermore, 0.5 wt% Pt loaded photocatalyst in an aqueous solution having 25% methanol showed the best hydrogen evolution rate *i.e.* 0.258 mmol h^−1^ g^−1^ and a photoconversion efficiency of 1.67%. Moreover, the reported enhancement in performance is due to the (i) formation of a large number of Ti^3+^, and (ii) doped sulphur forming the outer surface that broadens the photon absorption window, extending from UV to the near-IR region. Additionally, Fig. 17(a) presents the hydrogen evolution rate and Fig. 17(b) depicts the photocatalytic efficiency of the different sulfur-doped B-TiO_2_, respectively. The material could produce hydrogen for 20 h at a constant rate, and is stable up to 5 cycles without any significant change of catalytic performance, as given in Fig. 17(c). Furthermore, sulfur doping leads to the generation of vacancy states in the surface, causing the band tailing near the valence band edge. It also generates localized Ti^3+^ sites below the CBM, thereby narrowing the band gap, and increases the photocatalytic activity. Furthermore, the doped system shows excellent IPCE activity in the full UV spectrum (*i.e.* 74% (300 nm) and 84% (580 nm)), and even higher in the visible region (400 nm to 580 nm), which confirms the effective light harvesting ability of the catalyst due to the sulfide shell.^99^

**Fig. 18:**
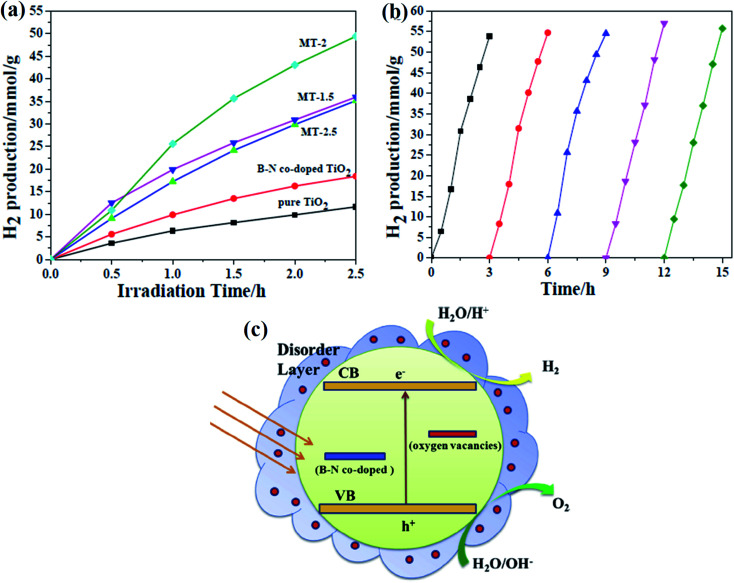
(a) Photocatalytic hydrogen production, (b) cyclic test of the photocatalyst towards hydrogen production, and (c) possible mechanism of the reaction taking place on the surface of the B–N co-doped TiO_2_. Reprinted with permission from ref. 100. Copyright 2019 Elsevier.

In another work, Wang *et al.* synthesized boron–nitrogen co-doped B-TiO_2_, where the team at first prepared B–N co-doped TiO_2_ by fast sol–gel technique using polyacrylamide and polyethylene as templates, and then these as-synthesized samples were subjected to a controlled magnesiothermal reduction process under N_2_ environment at 580 °C, followed by acid treatment, resulting in black TiO_2_. The material is composed of pure anatase phase, and the magnesiothermic reduction process does not cause any phase alternation (XRD analysis). Furthermore, the co-doped B-TiO_2_ possesses a crystalline core with surface-disordered amorphous shell containing plenty of oxygen vacancies. The performed Raman analysis indicates that the magnesiothermic reduction process brought about distortion in both lattice periodicity and octahedral symmetry of TiO_6_ on the outer surface. Moreover, the particles are uniformly distributed, having a particle size of 10–15 nm with Ti–N–B and Ti–N–B–O bonds confirming the doping of boron and nitrogen into the crystal lattice of the material. Additionally, the boron and nitrogen co-doping resulted in the formation of oxygen vacancies, which reduced the band gap due to the formation of defect energy states below the conduction band. In addition, under visible light irradiation, the photocatalyst showed excellent hydrogen production velocity *i.e.* 18.8 mmol h^−1^ g^−1^ in 20% aqueous methanol solution using Pt as a cocatalyst. The hydrogen generation rate is four times higher than that of pure white TiO_2_, and increases with an increase in the concentration of Mg during the magnesiothermic reduction process given in Fig. 18(a). Furthermore, the photocatalytic activity was explored for 15 h consisting of 5 cycles having a period of 3 h in each cycle, and no change in the rate of hydrogen production was observed, indicating the higher stability of the material, which is graphically represented in Fig. 18(b). The higher photocatalytic activity by the material is attributed to the formation of a Ti–N–B–O bond, which again indicates the presence of surface O–H groups that modify the surface structure and help in the effective separation of light-generated charge carriers. However, an interesting finding was reported by the author *i.e.* the evolution rate decreases after a certain Mg concentration (>2 : 1), but the light absorption capacity increases with an increase in the Mg content as the sample becomes darker. The anonymous behaviors are due to the formation of fresh recombination centers because of the excess reduction of TiO_2_ by Mg, which accelerates the exciton recombination, but enhances the photon capturing ability. The possible mechanism of hydrogen production is given in Fig. 18(c).^100^

**Fig. 19:**
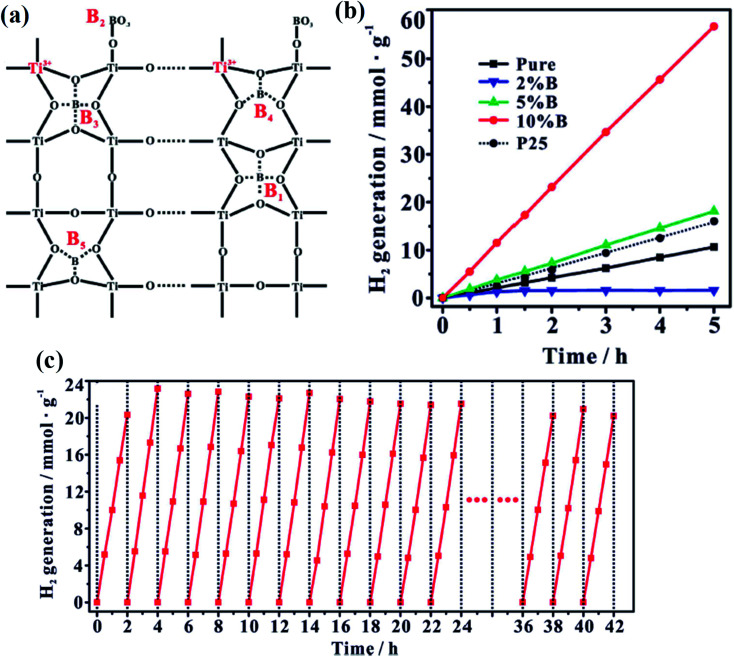
(a) Schematic illustrations of the boron sites, (b) photocatalytic activity, and (c) reusability test graph of 10% boron doped TiO_2_. Reprinted with permission from ref. 101. Copyright 2016 Nature Journal.

Moreover, Feng and his coworker have synthesized surface-disordered anatase TiO_2−*x*_ with a high amount of Ti^3+^ ions by doping boron into the crystal lattice *via* sol–gel method. The formed sample possesses a B–O–Ti linkage that helps in the formation of stable Ti^3+^ on the surface pictorially represented as Fig. 19(a). The band gap narrowing is due to the formation of Ti^3+^ impurities and disordered lattice introduced by boron doping, which extends the photon absorption capacity up to the IR region. The insertion of boron atoms into the TiO_2_ lattice generates oxygen vacancies and increases the dopant concentration. The lattice symmetry of TiO_2_ was damaged, which makes the lattice less symmetric *i.e.* disordered lattice. The interstitial B doping does not alter the crystal phase of the sample *i.e.* it remains in the anatase form, but the surface becomes disordered with a layer thickness of 1.5 nm encircling the crystalline core. Additionally, both theoretical and experimental studies illustrate that the doping of boron results in the formation of stable Ti^3+^ ions and surface structural disorder that ultimately affect the catalytic performance of B-TiO_2−*x*_. Along with the formation of a disordered surface, boron doping also created two mid-gap energy states at −3.8 to −2.0 eV and −1.4 to −0.5 eV, which lowers the band gap and increases the light absorption ability of the materials, resulting in enhanced photocatalytic activity. It was observed that with the increase in the dopant concentration, the photocatalytic hydrogen evolution rate increases. Among all prepared samples, 10% B–TiO_2_ showed the best result (*i.e.*, hydrogen evolution rate = 11.8 mmol h^−1^ g^−1^ and energy conversion efficiency = 21%) in the presence of Pt as a co-catalyst under 1 h of solar light illumination, which is about 3.5 times higher than commercial P25. No noticeable change in the photocatalytic activity was found, which proves its excellent stability (21 cycles and 42 h) and is represented as Fig. 19(b) and (c), respectively. Through this study, the author tries to explain the photocatalytic mechanism at the atomic level, which helps others working in this field in designing promising TiO_2_-based systems.^101^

**Fig. 20:**
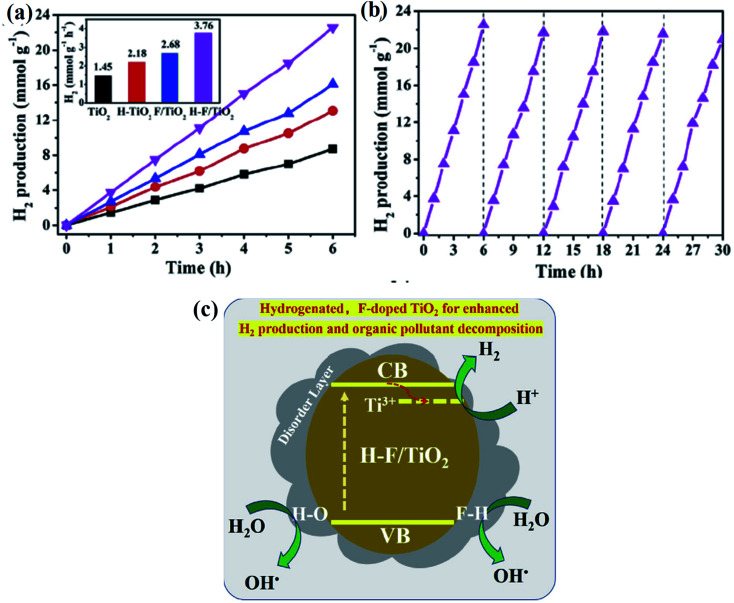
(a) The average rate of photocatalytic hydrogen production by different doped samples, (b) the stability of the material towards photocatalytic hydrogen generation, and (c) the proposed mechanism of the reaction occurring on the surface of the photocatalyst. Reprinted with permission from ref. 102. Copyright 2019 Elsevier.

Hydrogenated fluorine-doped black titania was prepared by Gao *et al.* by a facial two-step synthesis strategy taking titanium tetraisopropoxide as a precursor and NH_4_F as a fluorine source. Due to the high electronegativity of fluorine, it occupies the oxygen vacancy sites, producing a Ti–F bond. The material shows the presence of the anatase phase, where Ti is present in both +3 and +4 oxidation state. The particle size was about 20 nm in diameter with a 0.35 nm interplanar distance. Furthermore, the surface disorder layer is due to the O–Ti–F or F–Ti–F bond formed during the incorporation of the hydrogen species into the Ti–O linkage that enhances the surface hydroxyl groups, as well as the oxygen vacancies. Fluorine doping caused H–F and O–H bonds on the surface, which are excellent species for hole trapping, and reacts with water to generate a hydroxyl radical. In addition, the synergistic effect between the hydrogen insertion and fluorine doping leads to the broadening in the light absorption range. Furthermore, the hydrogenated fluorine-doped black titania showed an excellent hydrogen evolution rate of 3.76 mmol h^−1^ g^−1^ under visible light illumination, which is given in Fig. 20(a). This rate is 2.59 times higher than that of pure white titania, and 1.72 times higher than that of hydrogenated black titania. This photocatalyst also depicts extraordinary reusability *i.e.* 5 cycles up to 30 h, represented graphically in the form of Fig. 20(b), while Fig. 20(c) represents the possible mechanism for the reaction taking place on the surface of the photocatalyst.^102^

### 4.3 Metal as the co-catalyst

The photoresponse of a material can be increased by decreasing the rate of the charge recombination process, which could be done efficiently by transferring the photogenerated electrons to the cocatalyst matrix. The use of a cocatalyst also increases the active sites required for the proton reduction process. These active sites play an important role in decreasing the recombination rate. Usually, Pt can be used as a very good cocatalyst for photocatalytic hydrogen production. The Pt–TiO_2_ interface produces a Schottky barrier, which reduces the recombination rate of the charge carriers. However, due to its high hydrogen desorption energy, Pt shows a very low contribution toward the generation of hydrogen. Pt has an excellent ability to inhibit the back oxidation reaction of hydrogen to water, which reduces the overall catalytic efficiency of the material.

#### 4.3.1 Noble metal co-catalyst

**Fig. 21:**
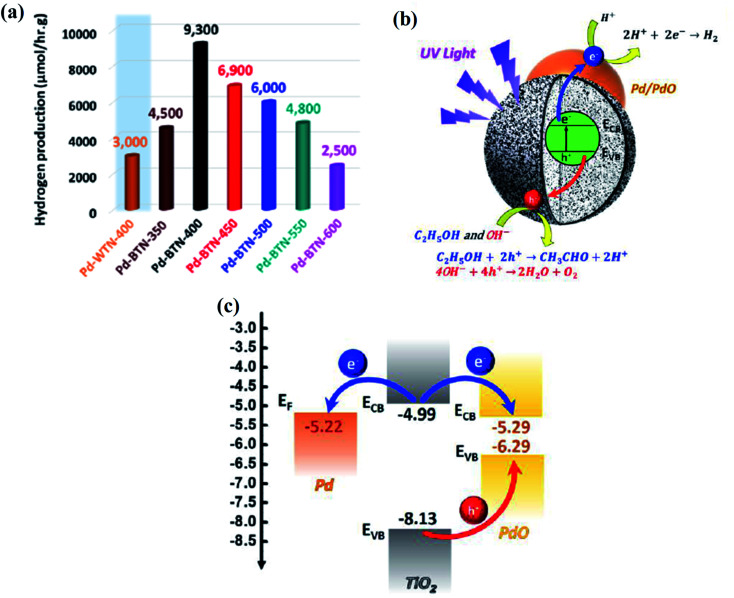
(a) The rate of hydrogen production by various wt% Pd-loaded black TiO_2_, (b) the possible mechanism occurring on the surface, and (c) the band structure of the material, along with the charge transfer process in Pd co-catalyzed black TiO_2_. Reprinted with permission from ref. 103. Copyright 2017 Elsevier.

In this context, Lian *et al.* have demonstrated a Ti–O bond by the reduction of Pt^4+^ on TiO_2−*x*_*via* an *in situ* fabrication method. The reduction of Pt^4+^ on the surface of TiO_2−*x*_ enhances the transfer of electrons from bulk to the surface, increasing the photocatalytic hydrogen evolution reaction. Furthermore, in another case, Zheng and the team fabricated a Pd–MgNi_*x*_ nanosphere and decorated it on a B–TiO_2_ porous film, which serves as an efficient photocatalyst for the proton reduction reaction. The cocatalyst Pd–MgNi_*x*_ can act as the oxidation and reduction sites, inhibiting the electron–hole recombination process, and can produce hydrogen at a rate of 34.93 mmol h^−1^ g^−1^.^98^ Additionally, Wu *et al.* reported rutile B-TiO_2_ by sol–gel method using titanium tetraisopropoxide as the Ti precursor, followed by thermal treatment in argon atmosphere. After that, Pd was deposited on the surface of B-TiO_2_ using the wet impregnation technique, which reduces the recombination rate of the photo-induced charge carriers. The crystal structure was found to be anatase, and the lattice ordering has been improved with an increase in the calcination temperature. The particle size of Pd has been estimated, and found to be 5.6 ± 0.8 nm with a ratio of Pd/Pd^2+^ at 53.9/46.1. The interface between the surface defects and Pd/PdO inhibits the electron and hole recombination, and thereby increases the lifetime of the charge carriers, leading to an excellent hydrogen production rate over the best catalyst *i.e.* Pd-BNT-400, which is 9300 μmol h^−1^ g^−1^ under the UV-B (*λ* = 312 nm, 8.0 W) and 5200 μmol h^−1^ g^−1^ in UV-A (*λ* = 352 nm, 8.0 W) light irradiation, as shown in Fig. 21(a). The higher photocatalytic activity is because of the surface of the material (Pd/PdO), which traps the photo generated electrons. Hence, this reduces the recombination and enhances the photocatalytic activity, and the mechanism of the electron transfer is shown in Fig. 21(b) and (c), respectively. Additionally, the presence of Ti^3+^, surface defect, Ti–O–H terminated bonding and oxygen vacancy makes a significant contribution toward the observed activity increment, as these sites act as charge trapping centers and prolong the lifetime of electron–hole pairs.^103^

#### 4.3.2 Non-noble metal as cocatalyst

**Fig. 22:**
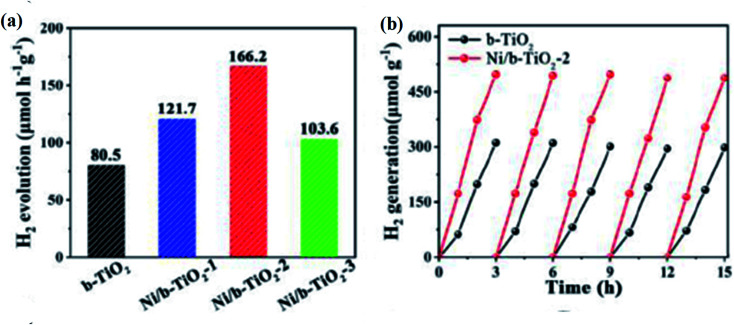
(a) Photocatalytic hydrogen generation rate by B-TiO_2_, Ni/B-TiO_2_-1, Ni/B-TiO_2_-2, Ni/B-TiO_2_-3, and (b) the reusability of the non-noble metal co-catalyzed B-TiO_2_. Reprinted with permission from ref. 105. Copyright 2020 Elsevier.

As noble metals have a high price tag and less availability, non-noble metals (like Ni, Co) can be used as an alternative co-catalyst for efficient photocatalytic hydrogen generation due to their low cost and appreciable activity under visible light. In this context, Lin *et al.* have fabricated a Ni and Co cocatalyst-modified B-TiO_2_*via* polymerized complex strategy, and examined its catalytic activity towards hydrogen generation under normal condition. The hydrogen production rate over TiO_2_ promoted by Ni and Co (0.1% Co and 0.2% Ni/TiO_2_) is about two times higher *i.e.* 2456 μmol (6 h) as compared to the monometal-loaded TiO_2_, *i.e.*, 1127 μmol H_2_ for 0.2% Ni/TiO_2_ and 1180 μmol for 0.1% Co/TiO_2_, respectively. The observed improvement in the H_2_ production of Co and Ni-loaded TiO_2_ is attributed to the presence of Co and Ni sub-band energy states, which widen the photon absorption capacity and reduce the exciton recombination process. Here, Ni and Co atoms are well dispersed on the surface of TiO_2_ as CoO and NiO, which play the role of the reaction site during the photoreduction and photooxidation process. Furthermore, the group evaluated the water reduction reaction of mechanically mixed TiO_2_ modified by Co and Ni *i.e.* 0.1% Co/TiO_2_ : 0.2% Ni/TiO_2_ = 1 : 1 (m/m) and observed a generation of 1282 μmol H_2_. In brief, the contact of NiO and/or CoO with TiO_2_ could effectively capture the photo-induced electrons and holes from the semiconductor, retarding the recombination rate. Moreover, by this experiment, the author tried to explain a facile method towards the development of promising non-noble metal-based cocatalystic systems for efficient H_2_ evolution reaction.^104^ Additionally, Ni nanosheets as cocatalyst on B-TiO_2_ was reported by Li and coworker using the hydrothermal method followed by high-temperature calcination. The B-TiO_2_ microspheres consist of both rutile and anatase phase, and as the concentration of nickel was increased, the material started showing absorption in both UV and Vis regions of the solar spectrum. The material is composed of uniform microspheres of TiO_2_ with a diameter of 1.3 μm, and the nickel nanosheets are placed on the surface of these spheres. Moreover, the material possesses a crystalline core–shell structure encapsulated by the nickel nanosheets, and the high-temperature hydrogenation process generated surface defects, oxygen vacancies, Ti^3+^ and surface –OH groups, which reduce the band gap and the material became visible light active. Ni/B-TiO_2_ showed excellent photocatalytic performance for hydrogen evolution *i.e.* 166.2 μmol h^−1^ g^−1^ represented in Fig. 22(a), which is 2 times higher than that of B-TiO_2_. The Ni nanosheet created a Schottky junction, which reduced the recombination rate of electrons and holes, and thereby enhanced the photocatalytic activity. Again, the photocatalyst does not show any noticeable change in its hydrogen production rate for 15 h, consisting of 5 cycles showing its higher stability (Fig. 22(b)).^105^

### 4.4 Heterostructure with B-TiO_2_

**Fig. 23:**
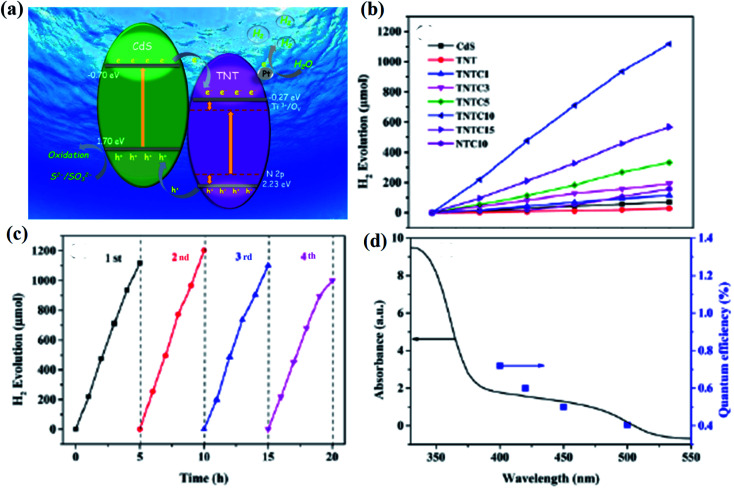
(a) Mechanism of hydrogen production by the composite TNTC10, (b) photocatalytic hydrogen generation rate by various samples, (c) reusability of the H–TiO_2_ composite, and (d) wavelength-dependent AQE of hydrogen evolution. Reprinted with permission from ref. 107. Copyright 2019 Elsevier.

Zhou *et al.* synthesized the heterostructure of Ti^3+^ self-doped mesoporous B-TiO_2_ with graphene *via* the solvothermal method. The composite with a narrower band gap and high surface area (68 m^2^ g^−1^) could absorb a broad range of visible light in the solar spectrum, which is represented pictorially as Fig. 23(a). The B-TiO_2_ in the heterostructure is composed of anatase phase with a band gap of 2.7 eV. Furthermore, the mesoporous black titania graphene heterostructure showed a hydrogen evolution rate of 186 μmol h^−1^, which is two times higher than that of mesoporous black TiO_2_ (96 μmol h^−1^). Graphene not only provides a platform for the mobility of electrons, but also provides numerous numbers of active sites for the photocatalytic reduction of the proton. As a result, the recombination rate is inhibited, which subsequently enhances the photocatalytic activity.^106^ Additionally, Qin and the team reported a binary heterostructure of nitrogen-doped B-TiO_2_ nanosphere with CdS nanorods *via* hydrothermal route. They found that the TiO_2_ part of the heterostructure is composed of pure anatase phase possessing Ti^3+^ as well as oxygen vacancies. The type-II heterojunction between the two could reduce the activation barrier for hydrogen production, and facilitates the excitons separation efficiency. Furthermore, the presence of mid-gap energy states due to oxygen vacancy in between the CB and VB results in the decrease of the band gap, and subsequently enhancing the photocatalytic activity. The strong electrostatic force of attraction between the CdS nanorod and nitrogen-doped B-TiO_2_ nanospheres initiates the charge transfer between the two. Out of the various wt% loaded CdS (1% to 15%), 10 wt% CdS-loaded B-TiO_2_ showed the highest photocatalytic activity with a hydrogen evolution rate of 1118.5 μmol, which is 40 times higher than that of nitrogen-doped B-TiO_2_. We also see that the material shows higher stability towards photocatalytic hydrogen generation for 20 h, consisting of 4 cycles, and does not show any appreciable change in the hydrogen production rate, as shown in Fig. 23(b) and (c), respectively. Furthermore, the wavelength-dependent apparent quantum yield is shown in Fig. 23(d).^107^

### 4.5 Nanostructured arrays of B-TiO_2_

#### 4.5.1 Active facet engineering

The property of the material greatly depends upon its size, shape, and structural states. Exposure of a particular facet/crystal plane of the catalyst can greatly influence the activity of the catalyst. From the stimulated computational study, it was concluded that different crystal planes or facets have different surface energies, resulting in different catalytic performances. In this direction, Chen *et al.* studied the effect of hydrogenation towards the photocatalytic hydrogen evolution reaction over the {001} facet of anatase TiO_2_ crystals, and demonstrated the correlation of the facet/morphology on the activity. In the hydrogenation process, F^+^ color sites and Ti^3+^ ions are the main defect center in the TiO_2_-{001} crystal, and are particularly positioned in the subsurface/bulk area. Similarly, in case of TiO_2_-{100}; F^+^, Ti^4+^–O˙ radical, Ti^3+^ and O_2_^−^ ions are observed from the surface to subsurface/bulk area, whereas for TiO_2_-{100}; F^+^, Ti^3+^ and the O_2_^−^ species are located in the same surface to subsurface/bulk region. These defect centers magnify the photon capturing and exciton formation ability of the nanocrystals, but at the same time, these sites act as a recombination center and slow down the charge separation process. However, the formed electric field between the stoichiometric surface and reduced/defective subsurface of the hydrogenated anatase TiO_2_ crystal containing a large amount of F^+^ color centers and Ti^3+^ (bulk) encourages effective charge carrier separation, which ultimately improves the hydrogen evolution rate to a significant level compared to the TiO_2_{001} nanocrystal. Furthermore, the H–TiO_2_-{100} and {101} catalyst show equal catalytic performance as that of TiO_2_-{100} and {101} nanocrystal, respectively.^108^ Furthermore, Zuo *et al.* reported a simple hydrothermal technique toward nucleation of non-stoichiometric rutile TiO_2_ with {111} and {110} facets by using Ti powder as a precursor, where the {111} and {100} facets act as charge carrier collectors, respectively, which enhances the separation of the photogenerated electron–hole pair. SEM and TEM analyses suggest that the TiO_2_ crystal is composed of both prismatic and bipyramidal phases *i.e.* {110} tetragonal prism and {111} tetragonal bipyramid with selective growth of {110} prismatic facet, which makes TiO_2_ stretch along the *c* axis. Furthermore, the reduction process generated Ti^3+^, which creates a vacancy state just below the conduction band, and results in a narrowing of the band gap. Reduced TiO_2−*x*_ with the exposure of the preferential facets shows much higher efficiency for water splitting as compared to TiO_2−*x*_ with irregular shapes *i.e.* 1 wt% Pt loaded photocatalyst could produce 1843 μmol of hydrogen during a period of 100 h with a turnover number of 1.47 and external quantum efficiency of 2.28% at 420 nm band pass filter. An interesting observation was made by the author *i.e.* loaded Pt nanoparticles specifically get attached to the {110} facet without any on {111}, respectively. The presence of Ti^3+^ as verified by EPR analysis plays a vital part in increasing the catalytic activity of the reduced TiO_2_ photocatalyst. Moreover, through this investigation, the group wants to demonstrate the effect of the chemical content and morphology on the catalytic efficiency of the material.^109^

#### 4.5.2 Ordered structures of B-TiO_2_

**Fig. 24:**
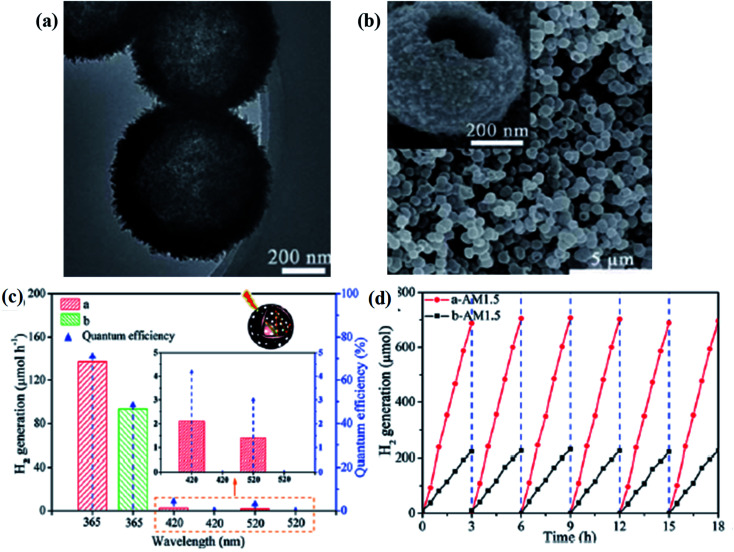
(a) TEM image and (b) SEM image of the stable mesoporous B-TiO_2_ hollow spheres, (c) photocatalytic hydrogen production rate by the mesoporous hollow spheres, and (d) cycling check for the stability of the mesoporous B-TiO_2_ hollow sphere. Reprinted with permission from ref. 110. Copyright 2013, Royal Society of Chemistry.

Moreover, mesoporous B-TiO_2_ hollow nanospheres can show excellent photocatalytic activity because of their hollow structure, which can effectively utilize solar light by providing many active sites. Hu *et al.* reported an assay synthesis technique for controllably preparing ultra-stable mesoporous hollow spheres of low bandgap B-TiO_2_ by a template-free facile two-step solvothermal process, along with amine molecule reflux-encircling method, followed by atmospheric hydrogenation. The formed mesoporous black TiO_2_ contains a large amount of Ti^3+^, surface disordered structure and high crystalline pore-wall. The surrounding ethylenediamine unit behaves as a protective shield, providing extraordinary thermal stability to the hollow framework, and prevents the grain nucleation and phase transformation, resulting in excellent structural stability. Furthermore, the hollow structure of the material was confirmed using TEM and SEM analysis, as the image is given as Fig. 24(a) and (b), respectively. The anatase form with hollow sphere morphology is stable up to a temperature of 900 °C without any crystal deformation. Additionally, the hollow spheres possess an impressive surface area of 80 m^2^ g^−1^, along with a pore size of 12 nm. Furthermore, the thickness of the wall and the diameter of the hollow spheres lie in between 1–500 μm and 35–115 nm, respectively. The reduction process leads to the generation of Ti^3+^ and the creation of oxygen vacancies, during which, the band gap was reduced to 2.59 eV from 3.17 eV, respectively. The 1 wt% Pt loaded mesoporous B-TiO_2_ hollow spheres showed a hydrogen evolution rate of 241 μmol h^−1^ 0.1 g^−1^, which is two times higher than that of B-TiO_2_ (118 μmol h^−1^ 0.1 g^−1^) and three times higher than that of mesoporous hollow spheres of pristine TiO_2_ (81 μmol h^−1^ 0.1 g^−1^), which is stable up to 6 photocatalytic cycles consisting of 18 h in total and is represented graphically as Fig. 24(c) and (d). The increment in catalytic performance is because of the high structural solidarity, improved crystallinity, high concentration of Ti^3+^ ions, and surface disorder that magnifies the photon absorption range and charge carrier separation efficiency.^110^

**Scheme 3:**
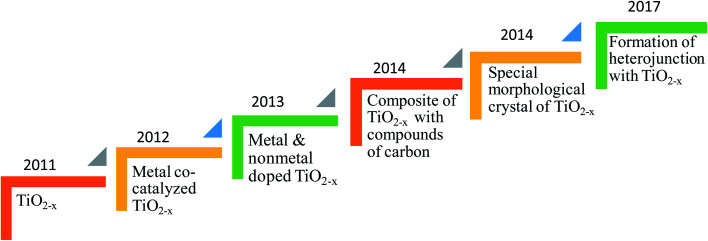
The timeline of black TiO_2_ progression for the productive hydrogen generation reaction.

The timeline of the development of B-TiO_2_ towards the photocatalytic hydrogen evolution reaction is schematically represented below (Scheme 3). Further, Table 1 depicts few reported B-TiO_2_ systems towards hydrogen evolution reaction.

**Table 1:** Various data obtained from the water-splitting reaction by using the photocatalyst (*i.e.*, B-TiO_2_) towards the hydrogen generation reaction

Sl no.	Sample notation	Conditions	Sacrificial reagent and co-catalyst	Irradiation source (wavelength)	Catalytic efficiency (%)	Rate of hydrogen generation	Durability	Reference no.
1	B-TiO_2_	20 mg of black TiO_2_ + 1 : 1 water–methanol solution	Methanol as a sacrificial agent and 0.6 wt% of platinum as cocatalyst	Full spectrum solar light	Energy conservation efficiency = 24	0.1 ± 0.02 mmol h^−1^ g^−1^	22 days	19
2	Pt-doped TiO_2−*x*_	50 mg of Pt doped TiO_2−*x*_ in 80 ml of methanol water solution (20 ml methanol & 60 ml water)	Methanol is used as a sacrificial agent	Four low-power 365 nm LEDs (3 W)	Quantum efficiency = 6.2%	151 μmol m^−2^ h^−1^	0.5 h	98
3	Sulphur-doped TiO_2−*x*_	100 mg of sulphur-doped TiO_2−*x*_ + 0.5 wt% Pt + 120 ml of 25% methanol solution	Methanol as sacrificial agent and Pt as cocatalyst	AM 1.5 simulated solar power system	Data not available	0.258 mmol h^−1^ g^−1^	20 h	99
4	Pt co-catalyzed black TiO_2−*x*_	50 mg of Pd/TiO_2−*x*_ in 2 liter mixture of ethanol and water in a ratio of 1 : 1	Methanol is used as a sacrificial agent	Sankyo Denki, G8T5BLB (*λ*_ma*x*_ ∼ 352 nm, 8.0 W)	Photon energy conversion efficiency = 4.12	5200 μmol h^−1^ g^−1^	Data not available	103
	Pd co-catalyzed black TiO_2−*x*_	50 mg of Pd/TiO_2−*x*_ in 2 liter mixture of ethanol and water in a ratio of 1 : 1	Methanol is used as a sacrificial agent and Pd as cocatalyst	Sankyo Denki, G15T8E (*λ*_ma*x*_ ∼ 312 nm, 8.0 W)	Photon energy conversion efficiency = 2.31	9300 μmol h^−1^ g^−1^	Data not available	
5	Ni co-catalyzed black TiO_2−*x*_	0.2 wt% Ni/0.1 g of the photocatalyst was taken in a methanol water mixture containing 10 ml of methanol in 500 ml of water TiO_2−*x*_	Methanol is used as a sacrificial agent and Pd as cocatalyst	250 W high pressure mercury lamp	Data not available	1127 μmol h^−1^ g^−1^	6 h	104
	Co co-catalyzed black TiO_2−*x*_	0.1 wt% Co/TiO_2−*x*_ 0.1 g of the photocatalyst was taken in a methanol water mixture containing 10 ml of methanol in 500 ml of water	Methanol was taken as a sacrificial agent and Co as co-catalyst	250 W high pressure mercury lamp	Data not available	1180 μmol h^−1^ g^−1^	6 h	
	Ni & Co co-catalyzed black TiO_2−*x*_	Mixture of 0.2 wt% Ni/TiO_2−*x*_+0.1 wt% Co/TiO_2−*x*_ 0.1 g of the photocatalyst was taken in a methanol water mixture containing 10 ml of methanol in 500 ml of water	Methanol was taken as a sacrificial agent and a mixture of Ni and Co as co-catalyst	250 W high pressure mercury lamp	Data not available	1282 μmol h^−1^ g^−1^	6 h	
6	Heterojunction of B-TiO_2−*x*_ with Ni nanosheets	The photocatalyst with a mixture of water and methanol	Methanol was taken as a sacrificial agent and Ni behaves as the cocatalyst	Stimulated sunlight irradiation with wave length 365 nm	Quantum efficiency = 43	166.2 μmol h^−1^ g^−1^	15 h	105
	Heterojunction of B–TiO_2−*x*_ with Ni nanosheets	The photocatalyst with a mixture of water and methanol	Methanol was taken as a sacrificial agent and Ni behave as the cocatalyst	Stimulated sunlight irradiation with wave length 420 nm	Quantum efficiency = 12	166.2 μmol h^−1^ g^−1^	15 h	
	Heterojunction of B-TiO_2−*x*_ with Ni nanosheets	The photocatalyst with a mixture of water and methanol	Methanol was taken as a sacrificial agent and Ni behaves as the cocatalyst	Stimulated sunlight irradiation with wave length 520 nm	Quantum efficiency = 6	166.2 μmol h^−1^ g^−1^	15 h	
7	Composite of black TiO_2−*x*_ with graphene	10 mg of the photocatalyst in a 100 ml mixture containing 80 ml water and 20 ml methanol	Methanol was taken as a sacrificial agent and 0.5 wt% H_2_PtCl_6_·6H_2_O as cocatalyst	Auto-light CEL-HXF300 Xe lamp (300 W)	Data not available	186 μmol h^−1^ 0.01 g^−1^	Data not available	106
8	{111} facet exposed B-TiO_2−*x*_	100 mg of the photo catalyst in 120 ml of methanol water solution (25 ml methanol and 95 ml water)	Methanol is used as sacrificial agent and 1 wt% of Pt as cocatalyst	300 W Xe lamp with a cut off edge at 400 nm	Turnover number = 1.47	18.1 μmol h^−1^ 0.1 g^−1^	100 h	109
9	TiO_2−*x*_ hollow nanospheres	100 mg of the photo catalyst loaded in 1 wt% of Pt placed in 100 ml methanol–water mixture in a ratio 1 : 4	Methanol is used as sacrificial agent and 1 wt% of Pt as cocatalyst	300 W Xe lamp with band pass filter of 365 nm	Quantum efficiency = 70	241 μmol h^−1^ 0.1 g^−1^	18 h	110
	TiO_2−*x*_ hollow nanospheres	100 mg of the photo catalyst loaded in 1 wt% of Pt placed in 100 ml methanol–water mixture in a ratio 1 : 4	Methanol is used as sacrificial agent and 1 wt% of Pt as cocatalyst	300 W Xe lamp with band pass filter of 420 nm	Quantum efficiency = 2.3	241 μmol h^−1^ 0.1 g^−1^	18 h	
	TiO_2−*x*_ hollow nanospheres	100 mg of the photo catalyst loaded in 1 wt% of Pt placed in 100 ml methanol–water mixture in a ratio 1 : 4	Methanol is used as sacrificial agent and 1 wt% of Pt as cocatalyst	300 W Xe lamp with band pass filter of 520 nm	Quantum efficiency = 1.4	241 μmol h^−1^ 0.1 g^−1^	18 h	
10	Nitrogen doped TiO_2−*x*_	50 mg of N doped TiO_2−*x*_ + 100 ml 8 : 2 water–methanol solution + 50 ml of 10 mg l^−1^ MO aqueous solution	Methanol as sacrificial agent and MoS_2_ as cocatalyst	300 W xenon-lamp equipped with a 420 nm cut-off filter	Data not available	1.882 mmol h^−1^ g^−1^	25 h	111
11	Boron-doped TiO_2−*x*_	0.005 g of the 10% B-TiO_2−*x*_ + 0.6 wt% of Pt	Methanol as sacrificial agent and Pt as cocatalyst	300 W xenon lamp	Photocatalytic efficiency = 21%	0.059 mmol h^−1^ g^−1^	42 h	112
12	Fluorine doped TiO_2−*x*_/MCF	0.20 g fluorine doped TiO_2−*x*_/MCF in 80 ml of 25% methanol aqueous solution with 2 ml H_2_PtCl	Methanol as sacrificial agent and Pt as cocatalyst	300 W Xe lamp	Quantum yield = 46% with hydrogen energy conversion efficiency = 34%	Data not available	12.5 h	113
13	AAr-TNT(B)	5 mg (2 cm^2^) sample with 3 wt% Pt, 100 ml of methanol/H_2_O solution (1/5 methanol/H_2_O)	Methanol as sacrificial agent and Pt as cocatalyst	300 W Xe arc lamp with a 400 nm cut-off filter	AQY = 13.4 (*λ* = 365 nm), photo flux/h^−1^ = 3.96 × 10^20^, light intensity = 10.38 mW cm^−2^	4.705 μmol cm^−2^ h^−1^	25 h	114
14	TiO_2_-BT-Au 10s	Required amount of sample dispersed in 10 ml of 30% methanol/H_2_O solution	10 ml methanol (30 vol%) sacrificial agent and Au as cocatalyst	300 nm Xe lamp fitted with 420 nm band-pass filter, intensity = 100 MW cm^−2^	AQY of 4.13% at 420 nm	34.37 μmol cm^−2^	18 h	115
15	Pt-loaded mesoporous TiO_2_-B nanobelts	70 mg of 0.5 wt% Pt-loaded mesoporous TiO_2_-B nanobelts were dispersed in the mixed solution (220 ml of H_2_O and 50 ml of CH_3_OH)	Methanol as sacrificial agent and Pt as cocatalyst	AM 1.5 light irradiation (*λ* > 300 nm, 100 mW cm^−2^)	Data not available	656.10 μmol h^−1^	Data not available	116
16	Ordered mesoporous black TiO_2_ (OMBT)	100 mg catalyst loaded with 1 wt% Pt suspended in a mixture of 80 ml H_2_O and 20 ml CH_3_OH	Methanol as sacrificial agent and Pt as cocatalyst	AM 1.5 with a power density of 100 mW cm^−2^	AQY of 62.3% at 365 nm	136.2 μmol h^−1^	30 h	117
17	Mesoporous Pt/NiS/black TiO_2_ hollow nanotubes (P-NBTNs)	50 mg catalyst loaded with 0.11 wt% Pt suspended in a mixture of 80 ml H_2_O and 20 ml CH_3_OH	Methanol as sacrificial agent and Pt as cocatalyst	AM 1.5 solar simulator with an AM 1.5G filter	Data not available	4.70 mmol h^−1^ g^−1^	24 h	118
18	10LBT/CdS	Sample 20 mg, 50 ml of sacrificial agent aqueous solution, *i.e.*, mixture of 0.35 M Na_2_SO_3_ and 0.25 M Na_2_S (50/50/v/v)	Na_2_S and Na_2_SO_3_ as sacrificial agent without any cocatalyst	300 W xenon lamp + *λ* ≥ 420 nm	Data not available	9.9 mmol h^−1^ g^−1^	16 h	119
19	Hydrogenated/nitrogen-doped black TiO_2_ nanoplates (NHTA)	100 mg catalyst loaded with 0.5 wt% Pt suspended in a mixture of 80 ml H_2_O and 20 ml CH_3_OH	Methanol as sacrificial agent and Pt as cocatalyst	300 xenon lamp (300 W) equipped with optical cut-off filters to realize AM 1.5	QE = 92% at 365 nm	1500 μmol g^−1^ h^−1^	15 h	120
20	Defective black TiO_2−*x*_(B) nanosheets	30 mg sample decorated with 0.03 wt% Rh were dispersed in 30 ml 10 vol% aqueous methanol solution	Methanol as a sacrificial agent and Rh as cocatalyst	A 500 W mid-pressure Hg lamp with 420 nm optical cut-off filters	Data not available	0.58 μmol g^−1^ h^−1^	Data not available	121
21	B-TiO_2_/g-C_3_N_4_ nano-heterojunctions	50 mg sample added to 10 ml triethanolamine and 90 ml DI water	Triethanolamine as sacrificial agent without any cocatalyst	300 W Xe lamp was used as the simulating sunlight source with intensity of 100 mW cm^−2^	Data not available	808.97 μmol g^−1^ h^−1^	30 h	122

## 5. Summary and future prospective

The utilization of renewable energy sources to address the ever-rising global energy demand, while keeping environmental purity intact, is the need of the hour. In this context, hydrogen fuel derived from the water *via* artificial photocatalysis is the most encouraging and clean strategy developed so far. Furthermore, in the hydrogen generation through water splitting method, both photocatalytic and photoelectrochemical routes under irradiation of sunlight were thoroughly studied by the scientific community. Metal oxide-oriented systems shows promising results, but the major associated bottle necks are the wide optical band gap, low exposed active site, slow charge diffusion, and rapid electron–hole recombination rate. However, the holy grain comes with B-TiO_2_, which plays a key role in photocatalysis, as it captures the maximum portion of solar radiation ranging from the UV to IR region, and it is the unique characteristic along with the defect site that promote charge separation. Additionally, with the presence of defective sites on the crystal surface, the adsorption and desorption of the reactants from the surface of the catalyst are facilitated. Since its discovery, B-TiO_2_ is a widely used catalyst for hydrogen generation by water splitting reactions with extraordinary results and stability.

B-TiO_2_ undergoes certain rearrangements like Ti^3+^, oxygen vacancies, surface hydroxyl groups and Ti–H (formed by doping of hydrogen), which modifies the electronic band structure and changes the color of the material from white to black. The band gap is reduced due to the band tailing (*i.e.*, mid gap energy states). Hence, the absorption of visible light is increased and the rate of the recombination of charge carriers decreases.

With all the advancements, some questions still arise, like which is the best method for the synthesis of the nanomaterial, and there is a doubtful explanation about the properties of the photocatalyst. Again, if the surface defect is responsible for the black color of the substance, then it does not explain why pure amorphous TiO_2_ is white. Some reports explain that Ti^3+^ and oxygen vacancies are the same, while some say that these two are different things. The mechanism for the transfer of an electron to the photocatalyst from the dopant sites should be discussed in detail with proper justifications. How the defective sites present in the bulk of the TiO_2−*x*_ affect the photocatalytic activities of the material and should be investigated efficiently. Despite the use of B-TiO_2_ as a photocatalyst, it can be used in lithium-ion batteries because of its higher lithium-ion storage capacity to increase the durability of the battery. It can also be used in supercapacitors. B-TiO_2_ also has biological applications in the field of cancer treatment. Although there are a lot of developments in the applications of B-TiO_2_, more studies are required for the use of this material in an efficient way to overcome the problems in human society. This review will encourage researchers to gain an idea about the synthesis and applications of B-TiO_2_ for photocatalytic hydrogen production, including state-of-the-art materials. Furthermore, the review discussed various characterization techniques used to justify the formation of B-TiO_2_ and the reason behind these observations. It also acts as a guiding tool for researchers working in the field of the supercapacitor, battery, phototherapy, and dye-sensitized solar cell, with B-TiO_2_ as the primary material to tune it accordingly for achieving benchmark efficiency. Additionally, this review will bring enthusiasm in their mind to create a facile and new approach for the fabrication of the photocatalyst with defective sites, along with enhanced photocatalytic activities.

## Conflicts of interest

There are no conflicts of interest to declare.

## Supplementary Material
